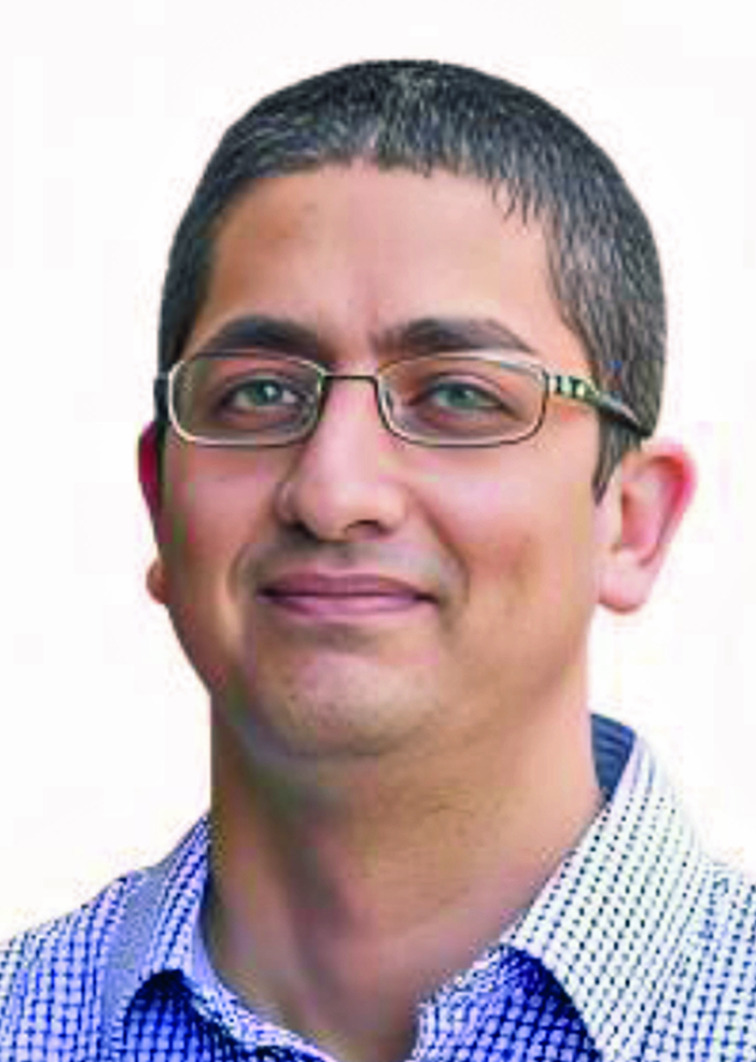# Thermochemical Recycling and Degradation Strategies of Halogenated Polymers (F−, Cl−, Br−): A Holistic Review Coupled with Mechanistic Insights

**DOI:** 10.1002/tcr.202500022

**Published:** 2025-04-07

**Authors:** Mohamed Shafi Kuttiyathil, Labeeb Ali, Mohammednoor Altarawneh

**Affiliations:** ^1^ United Arab Emirates University Department of Chemical and Petroleum Engineering, Sheikh Khalifa bin Zayed Street Al-Ain 15551 United Arab Emirates

**Keywords:** Halogenated polymers, De-fluorination, De-chlorination, De-bromination, thermal recycling

## Abstract

Handling the waste associated with halogenated polymers is a daunting task due to the well‐documented emission of halogen‐bearing toxicants during the disposal or recycling operation. According to the Stockholm Convention treaty, most of these products are classified as persistent organic pollutants due to their potential health hazards. This review aims to provide a holistic overview of the recent updates for treating halogenated polymeric waste through physical, chemical and biological approaches. In the line of inquiry, critical analysis of the obstacles and prospects associated with each degradation technique on the halogenated polymer has been performed, assessing based on the degradation efficiency, treatment upscaling, pollution control, and feasibility. Though many treatments show promising results, they also entail drawbacks. Thermal treatment exploiting various metal oxides, especially calcium additives, is considered the most executable technique for halogenated polymer valorization coupled with mineralization/metal extraction due to its intuitive operational feasibility and potential scalability. Strategies for combating the soaring halogenated polymeric wastes summarized herein tap into promoting a circular economy approach for their sustainable disposal and recycling

## Graphical abstract

This review aims to comprehensively address recent degradation techniques (physical, chemical and biological) of halogenated waste polymers with a prime focus on thermal degradation techniques. Exploiting various metal oxides during co‐pyrolysis is deemed a viable solution for recycling halogenated polymeric waste. This approach has multiple accomplishments of reducing toxic gas emissions and producing valuable products in addition to the invaluable metal recovery via water leaching from pyrochar.

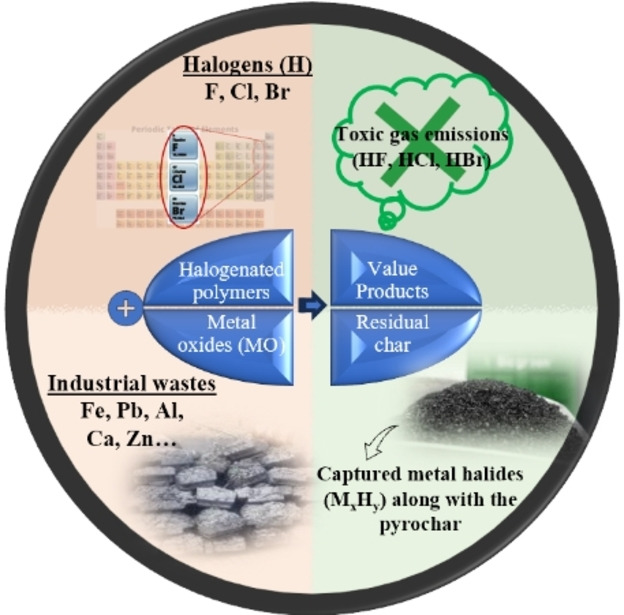

## 1 Introduction

Halogenated polymers (Fr‐, Cl‐, and Br‐)are known for their unique properties such as tensile strength, heat resistance, low conductivity, flame retardancy, liquid repellence, chemical and weather resistance. These properties make them invaluable in various applications across different industries, including construction, automotive, pharmaceutical, firefighting foam, electronics, textiles, kitchenware, food packaging, and protective coatings, spanning almost all vital sectors. Overseeing the growing population, the global annual waste generation is forecasted to exceed 3.4 billion tonnes by 2050.^[1]^ The rapid urbanization and associated industrial boom in each sector have resulted in serious halogenated polymer waste generation. The worldwide fluoropolymer production covers over 4700 chemicals under the per‐ and polyfluorinated alkyl substances (PFAS) category, which is estimated at 320,000 tons annually.^[2]^ Their application is mainly found in aqueous film‐forming foams as fire extinguishers, non‐stick coatings and liquid repellents.^[3]^ For chloro‐polymers such as polyvinyl chloride (PVC), it is estimated that global waste generation by 2025 is projected to rise by 0.9 billion tons annually.^[4]^ India and China lead the annual PVC global demand table, surpassing 50 million metric tons.^[5]^ By the end of 2050, the PVC trajectory analysis foresees that the accumulative PVC waste will be 508.6 Mt in China alone.^[6]^ Brominated polymers find their best application as a flame retardant and are mostly associated with the electrical and electronics industry. According to the United Nations in 2019, the average production from waste of electrical and electronic equipment (e‐waste) exceeded 54 million tons valued at $62.5 billion, of which proper recycling was done for only 17 %; the rest was either subjected to irrational treatment or ended up in landfills. E‐waste being the world's fastest‐growing solid waste stream, is expected to generate ~75 million tons by 2030.^[7]^

**Figure 1:**
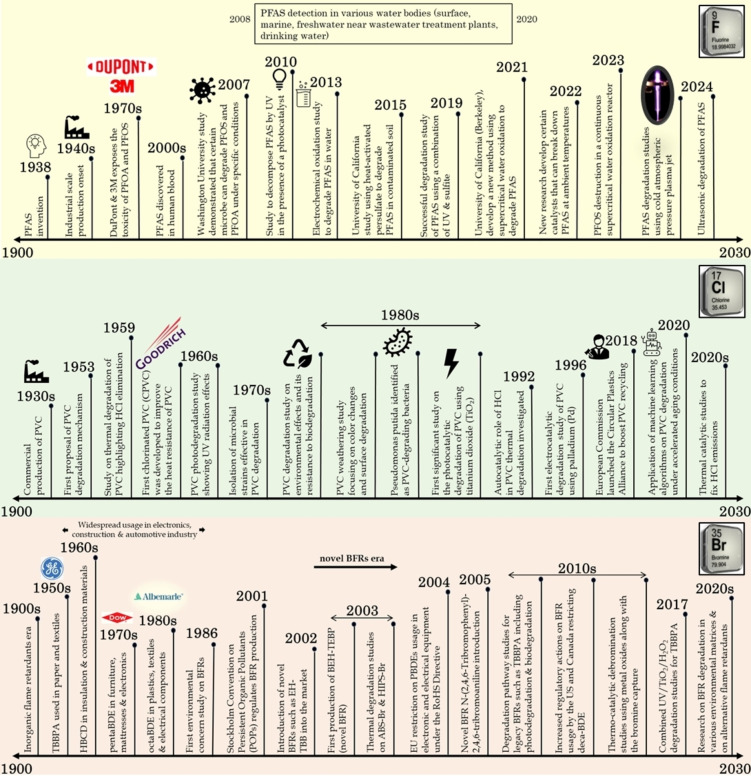
Historical timeline of the halogenated polymer degradation studies.

**Figure 2:**
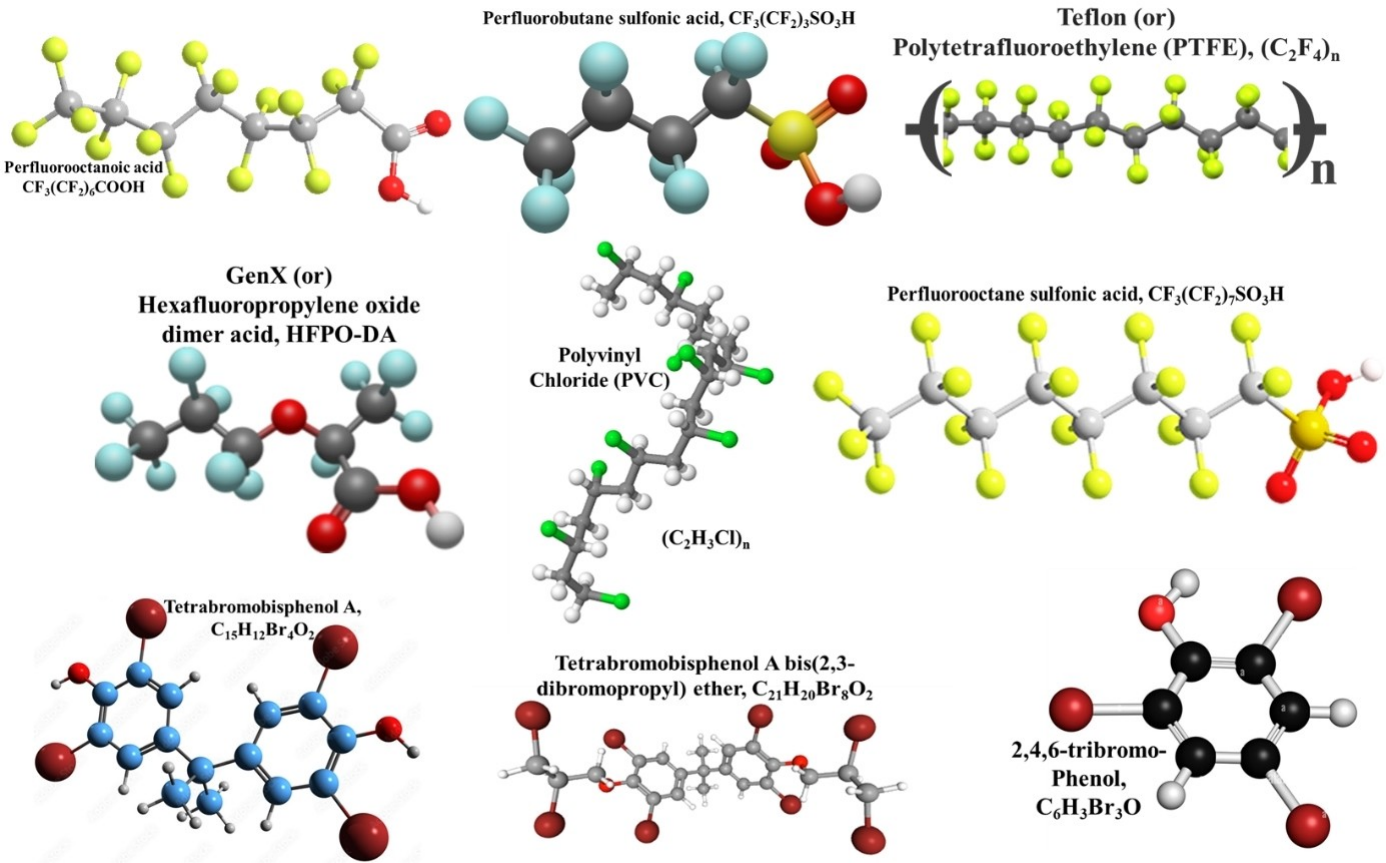
Potential halogenated polymers which are widely prominent in the environment.

The industrial‐scale production of PFAS started in the 1940s exploiting the presence of the most electronegative element (i. e., fluorine), attributed to its exceptional and significant physical characteristics. Owing to its extreme mobility, PFAS can penetrate various air and water matrices causing environmental menace. The recalcitrant carbon‐fluorine (C−F) bonds contribute to its propensity for bioaccumulation and its resistance to degrade in any typical municipal sewage treatment process and hence infamously known as ‘forever chemicals’.^[2]^ In the 1970s, DuPont and 3M company scientists exposed the toxicity of PFAS for the first time before discovering them in human blood in the early 2000s as shown in Figure 1.[^8^, ^9^] Since then, various PFAS degradation studies have been initiated as shown in Figure 1 leading into the latest perfluorooctanesulfonic acid (PFOS) destruction studies using supercritical water^[10]^, plasma treatment using cold atmospheric pressure plasma jet^[11]^ and ultrasonic degradation of PFAS.^[12]^ Recent PFAS exposure studies have linked it to various health issues such as adverse effects on immune function, thyroid disorders, liver disease, metabolic outcomes, and cancer[^3^, ^13^] and hence many of these chemicals are being added as persistent organic pollutants (POPs) to the Stockholm Convention's list. Since the 2000 United States Environmental Protection Agency (USEPA) press release, public anxieties over certain PFAS have grown due to their adverse environmental and health impact.^[14]^ PFAS can impact reproductive and developmental outcomes, cardiovascular health, and neurological function.^[15]^ While legacy PFAS like perfluorooctanoic acid (PFOA) and PFOS shown in Figure 2 have been controlled by regulations, the market intrusion by novel PFAS such as GenX or hexafluoropropylene oxide‐dimer acid (HFPO‐DA), 6:2 fluorotelomer sulfonamide alkylbetaine (6:2 FTAB) and hexafluoropropylene oxide‐trimer acid (HFPO‐TA) is inevitable with higher genotoxicity and endocrine‐disrupting effects in humans in addition to 6:2 fluorotelomer unsaturated carboxylic acid (6:2 FTUCA) and 6:2 fluorotelomer sulfonic acid (6:2 FTS).^[16]^ The expulsion of highly corrosive hydrofluoric acid (HF) during various degradation techniques and their conversion into ultrashort‐chain PFAS challenges the researchers in their combating studies as they accumulate at ~100x higher concentrations in the environment.^[17]^ Greenhouse gases including tetrafluoromethane (CF_4_) and hexafluoroethane (C_2_F_6_) were produced during PFOS combustion, which has long lifetimes of 50,000 and 10,000 years and exhibits global warming potentials of 5,700 and 11,900 respectively.^[18]^ The recalcitrant nature of PFAS either in the long‐chain or short‐chain form coupled with the significant health hazards posed by them drives researchers to find solutions to permanently remove them from different environmental matrices safely.

Chlorinated polymers such as chlorinated polyethylene, chloro‐sulfonated polyethylene, chlorinated polypropylene, chlorinated PVC and polychloroprene (neoprene) are versatile polymers typically ranging from 30–68 wt.% chlorine depending upon the applications.^[19]^ They impart various desirable properties such as improved rigidity, insulation, flame resistance, chemical resistance, toughness, flexibility and weatherability by virtue of the incorporation of chlorine atoms displacing the hydrogen atoms. The same properties hinder the hassle‐free management of the chlorinated polymeric wastes generated. Advancement in the construction industry and the high tensile strength of the thermoplastic, PVC led to its commercialized production in the 1930s as shown in Figure 1. PVC provides a better insight into possible end‐of‐life consequences for chlorinated polymers, as the fate of other chlorinated polymeric wastes is rarely studied.^[20]^ In certain European countries, though the waste PVC goes to waste‐to‐energy plants, the hydrogen chloride (HCl) toxication during the incineration process deteriorates the facility and it tends to be a menace for waste handlers.^[21]^ Approximately 90 % of waste PVC gets disposed of in the environment since only 10 % of it is regularly recycled. Due to their huge space requirements and lack of biodegradability, certain waste PVCs are being dumped in landfills worldwide, which is unacceptable.^[22]^ According to PVC recycling measures, there are serious environmental issues because of the lengthy degradation period and the toxicity of the breakdown products. Hence, in December 2018, the European Commission launched the Circular Plastics Alliance (Figure 1) to foster effective PVC recycling.^[23]^ Incineration of PVC can lead to the emission of hydrochloric acid (HCl) and other highly toxic/dangerous chloro‐organic compounds such as polychlorinated dibenzo‐*p*‐dioxins and dibenzofurans (PCDD/Fs), especially in the presence of aromatic compounds due to their high chlorine content of ~58 %.[^22^, ^24^, ^25^, ^26^] The traditional landfilling disposal methods cause potential environmental contamination.^[27]^ However, recent innovative approaches in managing chlorinated plastic waste include tandem catalysis for converting the chlorine‐containing waste into valuable aryl chlorides.^[27]^ Various upcycling strategies leverage or overcome the C−Cl bond through depolymerization, carbonization, and modification techniques.^[20]^ Researchers worldwide are actively investigating several innovative ways to accelerate PVC decomposition safely and to diminish its detrimental effects.

Albeit offering excellent flame‐retardant properties to various consumer products including plastics, electronics, textiles and building materials; brominated polymers have raised environmental concerns due to the potential release of harmful brominated compounds during their degradation. During the 1950s, tetrabromobisphenol A (TBBPA) in the paper and textiles industry, hexabromocyclododecane (HBCD) in the insulation and construction industry during 1960s (Figure 1) and decabromodiphenyl ether (DBDE) were the leading brominated flame retardants (BFRs) used followed by pentabromodiphenyl ether (pentaBDE) and octabromodiphenyl ether (octaBDE).[^28^, ^29^, ^30^] BFRs in e‐waste pose significant challenges for recycling and waste management. The environmental studies indicate that BFRs are ubiquitous in sediment and biota, with increasing levels of some compounds potentially causing adverse effects on wildlife and humans. Though restrictions have already been sanctioned on legacy BFRs such as TBBPA after the Stockholm Convention in 2001 (Figure 1), the novel BFRs intrusion into the market is highly alarming.[^31^, ^32^] Like other polymers, BFRs also end up in incineration facilities or landfills, creating environmental hazards by releasing aerial toxins and aquifer contaminations. Studies have explored various techniques to detect and safely capture toxic hydrogen bromide (HBr), polybrominated dibenzo‐*p*‐dioxins and dibenzofurans (PBDD/Fs) from BFRs during the degradation process.[^25^, ^33^, ^34^] The short‐wave infrared spectroscopy has shown promise in identifying brominated plastics with 89–90 % accuracy.^[35]^ Approximately 2200 tons/yr of waste exceeds the low persistent organic pollutants (POPs) concentration limits for polybrominated diphenyl ethers (PBDEs) and HBCD in Ireland, demanding a distinctive treatment.^[36]^ Poor recycling practices have led to BFR contamination in children′s toys and food‐contact articles, with 61 % of the samples examined testing positive for bromine presence.^[37]^ In animals, BFRs may disrupt the thyroid hormone homeostasis and stimulate neurobehavioral changes.^[38]^ Usually, the BFRs persist in the ecosystem and thereby bio‐accumulate in humans due to their lipophilicity causing potential adverse health effects such as cancer, reproductive problems, and metabolic disorders.^[39]^ BFRs are also anticipated to induce epigenetic modifications, such as global DNA hypomethylation, changes in histone modifications, and also altering endocrine functions, potentially contributing to genomic instability and developmental issues.^[40]^ The primary concern appears to be endocrine disruption, with hepatotoxicity and neurotoxicity also being significant.^[41]^ The novel BFRs presence in feeding‐mothers′ milk was reported in China^[42]^, Ireland^[43]^ and various other studies. Improved sorting and treatment techniques are crucial to prevent BFRs from entering the recycling stream, contaminating revived products and capturing toxic emissions such as HBr and PBDD/Fs.

**Figure 3:**
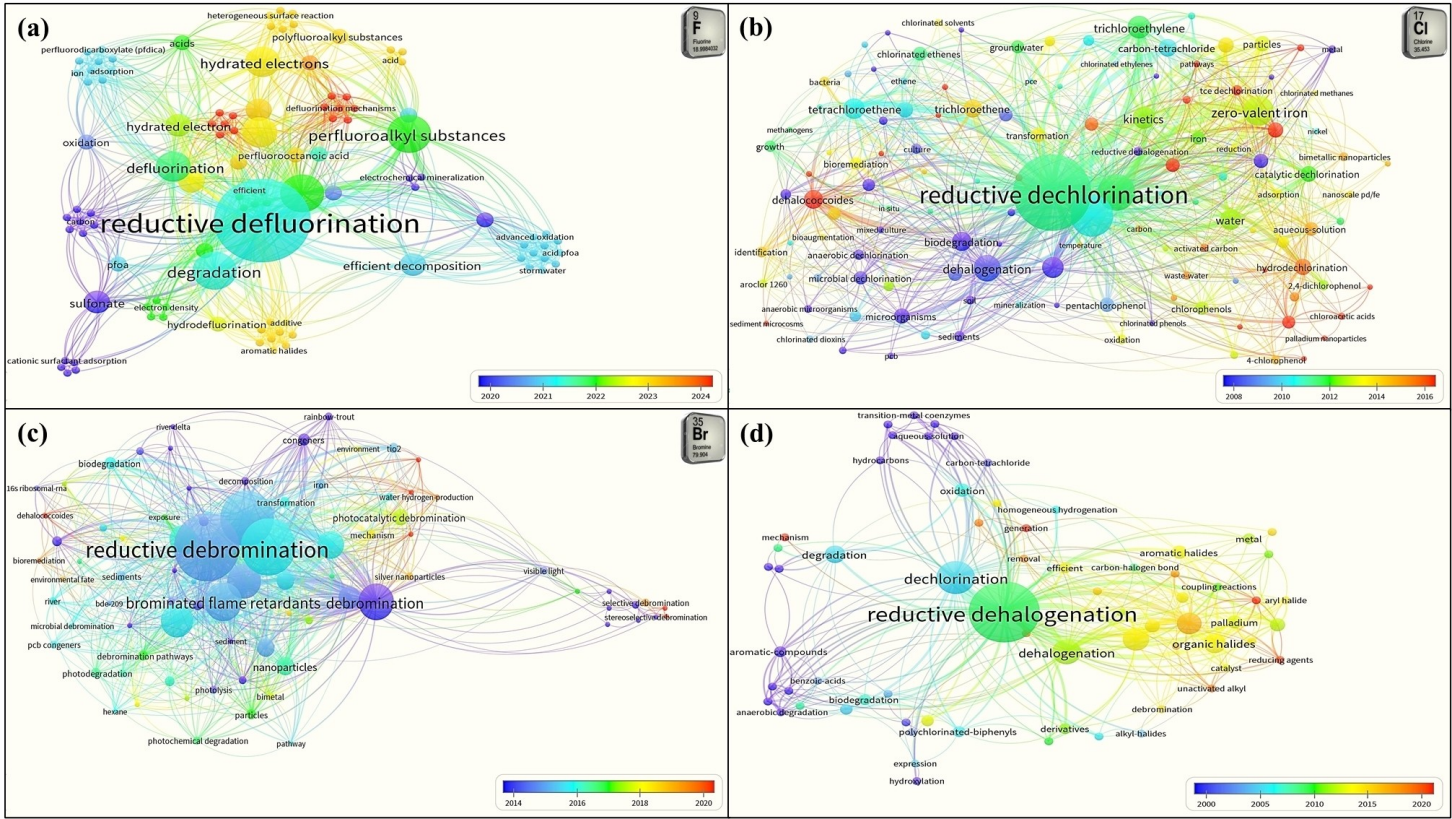
Bibliometric analysis of the most‐used keywords in articles on (a) de‐fluorination, (b) de‐chlorination, (c) de‐bromination and (d) dehalogenation.

While visualizing the scientific studies done over the past couple of decades on dehalogenation research using VOSviewer as shown in Figure 3, reductive reactions were found to be more prominent wherein the halogens are replaced by H atoms in any degradation process. A visual map was created based on the bibliographic data from the Web of Science core database for the ‘Co‐occurrence’ of ‘All keywords’ upon a ‘Full counting’ basis in ‘Title’, ‘Abstract’ and ‘Keywords’ which met a minimum threshold of at least 5 times the keyword being occurred. The most commonly used keywords by authors worldwide were analyzed separately. Upon analysis, the keywords “reductive defluorination” in Figure 3(a), “reductive dechlorination” in Figure 3(b), “reductive debromination” in Figure 3(c) and “reductive dehalogenation” in Figure 3(d) appeared with the most link strength in respective cases. PFAS was the second most frequently occurring keyword in defluorination studies, zero‐valent iron in dechlorination studies and BFRs in debromination studies. While defluorination research panned studies from 2020 to 2024, dechlorination started studies from 2008, whereas debromination from 2014. However, the dehalogenation studies panned from 2000 to 2020 with emphasis on the reductive reactions to displace halogens. The objective of this review is three‐fold: (i) to abridge the most recent biological, chemical and physical treatment techniques for recycling/upcycling the halogenated wastes, (ii) to understand the conversion and the mechanistic pathways pertaining to the co‐pyrolysis of halogenated wastes with metal oxides (MOs), and (iii) to suggest the potential characterization techniques probably useful while conducting a halogenated waste upgradation study. This review intends to provide a brief overview of the likely plausible improvement in the current degradation studies on a halogenated polymer by providing insight into the degradation mechanism of halogenated waste conversion into valuable products. The augmentation in the current recycling strategies is anticipated to promote carbon neutrality and circular economy to meet the United Nations (UN) Sustainable Development Goals (SDGs) such as SDG #7 and SDGs #12–15. The novelty in this review is to portray the most recent updates (within 5 years) in the halogenated waste upgradation techniques targeting the most convenient thermal conversion pathways to unearth the potential challenges and prospects.

## 2 Halogenated Polymer Degradation Approaches

### 2.1 Fluorinated Polymers

**Figure 4:**
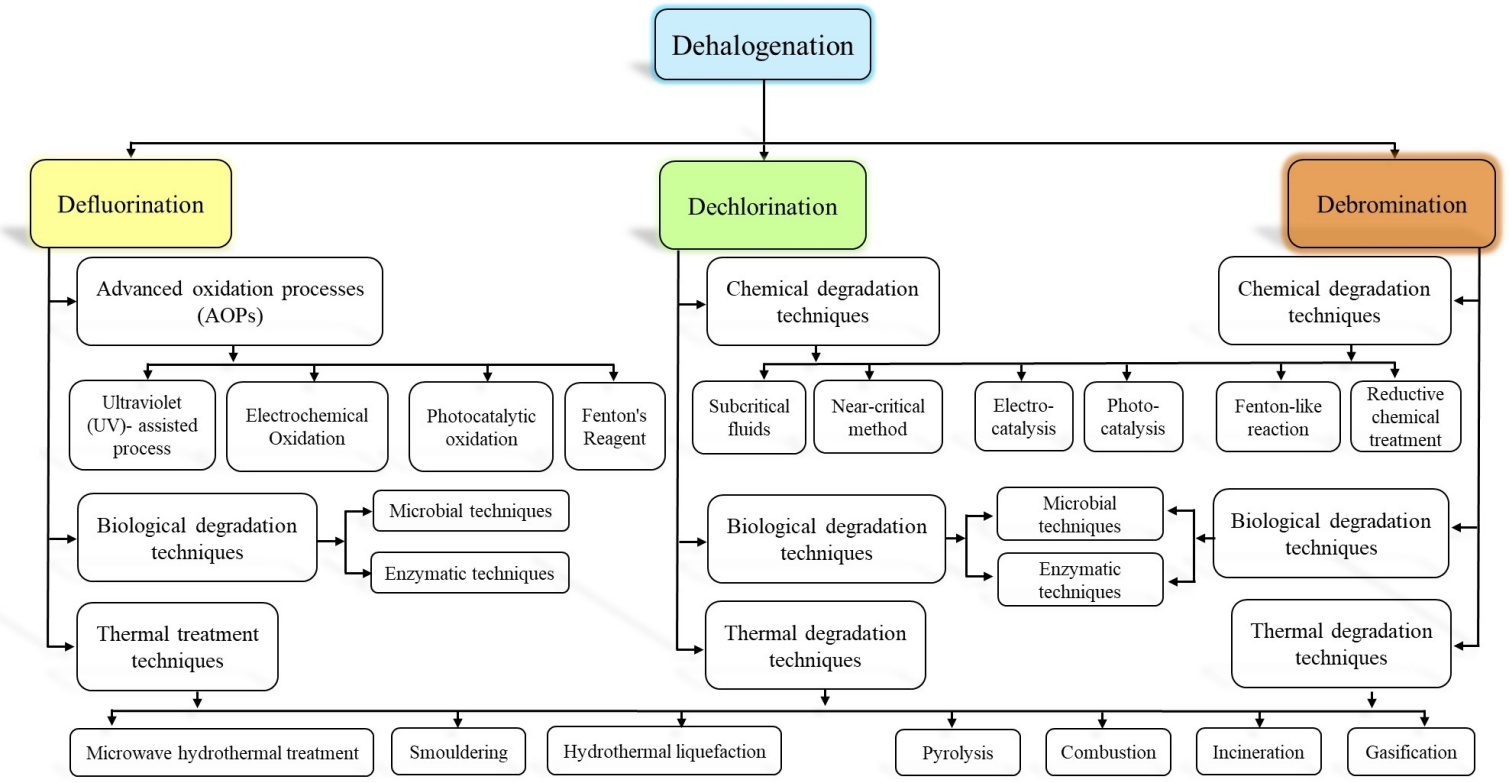
Major dehalogenation treatment techniques discussed in the literature.

The PFAS is posing a significant environmental concern due to their widespread use in multiple industrial sectors owing to their unique properties discussed before, such as resistance to heat, water, and oil. Characterized by their strong C−F bonds and unique properties, these “forever chemicals” are resistant to degradation and extremely persistent in the environment. Defluorination is more difficult than oxidation‐based C−C chain degradation unless the treatment targets the C−F bond specifically. The recent PFAS degradation techniques are being explored herein as shown in Figure 4, delving into both traditional and emerging methods. PFAS degradation in different environmental media is extremely challenging due to their persistence; the highly stable structure of PFAS is attributed to the high C−F bond dissociation energy (BDE) which is 485–540.0 kJ/mol.[^44^, ^45^, ^46^] The H‐atoms of the hydrocarbon chain are fully substituted with F‐atoms giving superior stability to the PFAS molecule leading to its bio‐accumulation.[^47^, ^48^] Hence, biological treatment and activated carbon adsorption in wastewater treatment methods are ineffective in removing these PFAS, thus mitigating the PFAS to migrate into other environmental matrices.

#### 2.1.1 Advanced Oxidation Processes (AOPs)

The AOPs are the promising technologies and also the most studied methods for PFAS degradation wherein the generation of highly reactive species, such as hydroxyl radicals (⋅OH) is involved, capable of oxidizing and degrading PFAS.

##### 2.1.1.1 Ultraviolet (UV)/Hydrogen Peroxide (H_2_O_2_)

While the UV photolysis has shown some efficacy, the augmentation with H_2_O_2_ to produce ⋅OH does not significantly enhance PFOA degradation, suggesting that alternative mechanisms should be explored.^[49]^ Introducing carbonate radical anions (CO_3_⋅^−^) to the UV/H_2_O_2_ process exhibited 100 % PFOA decomposition.^[50]^ Using reductants like sulphite and iodide, UV‐assisted advanced reduction processes (UV‐ARPs) have gained attention^[51]^ for their ability to convert PFAS into non‐fluorinated organics and fluoride ions.^[52]^ However, the presence of dissolved oxygen and organic matter can decrease the effectiveness of UV‐ARPs, necessitating pre‐treatment and anoxic conditions for optimal results.^[52]^ Overall, this process can also be limited by the penetration depth of UV light in the water matrix. Moreover, due to the competitive absorption of photons and competitive ⋅OH reactions with most hydrocarbons in water than with PFOA, H_2_O_2_ addition isn't considered wise for PFOA degradation in water as the maximum second‐order rate constant of *k*⋅_OH_+PFOA is only ≤10^5^ L/mol/s.^[53]^

##### 2.1.1.2 UV/Titanium Dioxide (TiO_2_)

The renowned photocatalyst TiO_2_ can absorb UV light and generate electron‐hole pairs, leading to the formation of ⋅OH radicals which could be used to degrade PFAS in both water and soil, particularly PFOA and PFOS. The vacuum ultraviolet (VUV)/TiO_2_ photocatalysis in acidic conditions (pH 4) with HClO_4_ has demonstrated effective PFOA degradation along with toxicity reduction.^[54]^ Even at extremely acidic conditions (pH 2), the biosynthesized TiO_2_ nanoparticles under UV/VIS (visible) light achieved a defluorination of 56.13 % and 95.62 % degradation efficiency for PFOS.^[55]^ The effectiveness of any UV‐based processes depends on the factors such as pH, water quality, and irradiation source. In lab‐scale studies, low‐pressure Hg lamps are commonly used, emitting at 254 and 185 nm. While UV/TiO_2_ techniques show potential, challenges still exist in understanding the defluorination mechanisms, the impact of co‐existing ions, and post‐treatment toxicity.^[53]^

##### 2.1.1.3 Fenton's Reagent

Fenton's reagent is an innovative mixture of the ferrous ions (Fe^2+^) and H_2_O_2_ which generates ⋅OH radicals through a complex reaction mechanism. A UV‐Fenton reaction catalyzed by nanosized Fe_3_O_4_ demonstrated ~90 % degradation efficiency for PFAS in an alkaline medium.^[56]^ A hybrid process combining Fenton reagent with humic acids completely immobilized PFOA from water at 25 °C and 100 min reaction time by entrapping it in the solid phase during humic acid oxidation.^[57]^ Leung and coworkers^[58]^ endorsed that ⋅OH could disrupt PFOA with limiting factors such as the Fe^2+^ concentration (initial), H_2_O_2_ concentration and solution acidity in the Fenton's reagent. However, this process is barely capable of degrading PFAS and is also limited by iron sludge generation.

##### 2.1.1.4 Electrochemical Oxidation

The electrochemical degradation using boron‐doped diamond (BDD) electrodes has emerged as a promising technique for treating water contaminated with PFAS.[^59^, ^60^, ^61^, ^62^] Uwayezu and coworkers^[63]^ degraded up to 56 % of PFAS in wastewater and achieved efficient PFOA degradation up to 99.5 % and fluoride generation up to 50 % by using BDD. This method is believed to effectively degrade the PFAS into shorter‐chain analogues and ultimately achieve mineralization even in the presence of natural organic matter.^[64]^ The key factors that can influence the PFAS degradation include current density, treatment time, anodic material, cathodic material and initial PFAS concentration, which could be inhibited by the presence of natural organic matter and high chloride content. The recent progress in electrochemical oxidation is primarily focused on the optimization of the anode materials, reactor design, and process parameters.^[65]^ Even though it is applicable for large‐scale applications due to its simplicity and low chemical loading, challenges like the formation of inorganic by‐products (perchlorate) remain.[^59^, ^65^] Though it is a process that can be effective for degrading PFAS, it requires significant energy input. Though AOPs can be considered as promising technologies and are effective in breaking down a wide range of PFAS compounds by themselves, they could also be combined with other treatments for enhanced efficiency. However, the high energy demand and operational costs along with the toxic by‐product formation could be challenging.

#### 2.1.2 Microbial Degradation Techniques

**Figure 5:**
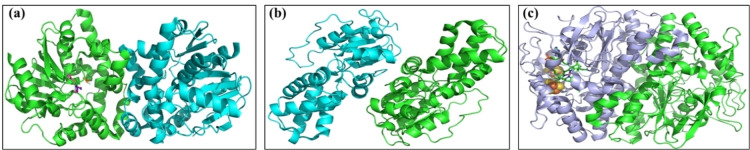
Potential enzymes for the biotransformation of PFAS via induced reactions (a) fluoroacetate defluorinase, (b) haloacid dehalogenase and (c) reductive dehalogenase. All the images are reproduced with permission.^[66]^ Copyright © 2024, American Society for Microbiology licensed under CC BY 4.0.

The microbial degradation techniques for PFAS as tabulated in Table 1 are the emerging approaches to address these persistent environmental contaminants. As the technique exploits the ability of the microbial strains and specific enzymes to break down PFAS molecules, it is considered an eco‐friendly approach. Several enzyme‐induced reactions for the biotransformation of PFAS have shown potential for its degradation including haloacid dehalogenases, peroxidases, cytochromes, fluoroacetate defluorinases, alkane monooxygenases, reductive dehalogenases, butane monooxygenases, desulfonases and laccases as shown in Figure 5.[^66^, ^67^] Although microbial degradation offers an environment‐friendly solution, the number of identified microorganisms capable of transforming the PFAS remains limited.^[66]^ Rational *in silico* enzymatic design was explored by Marciesky and coworkers^[68]^ to enhance the PFAS degradation capabilities using the radical generating enzymes, *S*‐adenosylmethionine and Adenosyl cobalamin containing horseradish peroxidase and laccase. *Delftia acidovorans* dehalogenase has exhibited its potential for enzymatic defluorination of a fire‐fighting foam‐loaded soil comprising PFOA/S contaminations.^[69]^ Though various PFAS treatments exist, enzymatic and microbial approaches are considered more sustainable and cost‐effective due to their low energy requirement.^[70]^ However, due to the limited knowledge of the enzyme‐PFAS interaction know‐how, the scalability and stability of enzymes in various environmental conditions make it challenging for researchers.

**Table 1:** Various PFAS treatment approaches ‐ recent studies (within 5 years).

Methods	PFAS targeted	Treatment conditions	Defluorination efficiency (DF)/ Degradation efficiency (DE)/ Removal efficiency (RE)	References
Photodegradation	PFCA, FTUCA and GenX	KrCl* excimer lamp, direct irradiation at 222 nm for 4 hours.	PFCA (24 %), FTUCA (31 %), GenX (17 %) (DF)	^[79]^
	PFOS	150 min UV irradiation, pH 2, biosynthesized TiO_2_ nanoparticles.	56.13 % (DF)	^[55]^
	PFOA, PFBS, PFHxS, GenX, and 6 : 2 FTS	Photocatalytic ozonation (10 mg O_3_ L^−1^) under UVA‐visible radiation, 5 wt.% WO_3_/TiO_2_ catalyst.	6 : 2 FTS (24 %), PFOA (23 %), PFHxS (19 %), PFBS (9 %), GenX (4 %) (DF)	^[80]^
	PFOS	450 W UV lamp, 30 min mechanical stirring, *f*‐TMO hybrid catalyst.	67.6 % (DF)	^[81]^
	PFOA	450 W UV lamp, 30 min mechanical stirring, *f*‐TMO hybrid catalyst.	74.8 % (DF)	^[81]^
Electrochemical	PFOS, PFOA, PFHxS, PFHxA, PFBS and PFBA	Graphene sponge anode, 1 mS.cm^−1^ electrolyte, 230 A/m^2^ current density, 10.1±0.7 kWh/m^3^ energy.	PFOS (24.0 %), PFOA (21.8 %), PFHxS(14.5 %), PFHxA (8.5 %), PFBS (11.7 %), PFBA (8.1 %) (DF)	^[82]^
	PFOS, PFHxS and PFOA	10 mM Na_2_SO_4_ and 2 mM NaCl electrolyte, electrochemical oxidation, BDD anode, room temperature, 1.8‐40 mA/cm^2^ current density.	89.1 % PFOS, 88.1 % PFHxS 94.0 % PFOA (RE)	^[60]^
	PFOA	SPEF system with MOFs/CNF cathode, −0.2 to −0.6 V under O_2_ purge, 3 hrs.	59 % (DF)	^[83]^
	PFOA	BDD anode, redox copolymer /CNT cathode, DI water + 20 mM NaCl electrolyte, 5 hrs.	80 % (DF)	^[84]^
	PFOS	BDD/SnO_2_‐F anode, 1 M NaCl electrolyte, 30mA/cm^2^ current density, 30 mins.	61.39 % (DF)	^[61]^
Thermal	PFSA, PFCA	PFAS in soil, sealed/ horizontal reactor in air/ N_2_,≥400 °C, 30 mins.	PFSA 60−71 %, PFCA ~99 % (DE)	^[85]^
	PFOA, PFOS	PFAS‐impacted biosolid, pilot‐scale pyrolysis reactor (63.6 kg/h), residence time 19.1 mins, 649 °C.	mean >97.4 % in biochar (RE)	^[86]^
	PFHxA, PFNA, PFOA, PFPeA, PFUnDA	PFAS‐contaminated biosolid, bench‐scale mechanically fluidized pyrolysis reactor, feed rate 25 g/min, 700 °C.	88.2 wt.% in biochar (RE)	^[87]^
	22 target PFAS	PFAS‐impacted biosolid, slow pyrolysis, 10–15 °C/min under Ar (10–15 mL/min), 20 min, 500, 650 and 800 °C.	>99 % in biochar (RE)	^[88]^
	PFBS, PFHxS, PFHpA, PFOA, PFOS, PFNA	PFAS‐contaminated GAC/ soil, 5 cm/s air flux, avg. smouldering velocity 0.51 cm/min, >900 °C.	44 % (GAC), 16 % (soil) (DE)	^[89]^
Biodegradation	TFA, PFOA and HFPO‐DA	Anaerobic microbial consortium, proteobacteria and bacteroidetes, room temperature (~22 °C), pH ~7.2, 10 months incubation.	TFA 8.03 %, PFOA 13.52 %, HFPO‐DA 5.45 % (RE)	^[90]^
	PFOA, PFOS	Aerobic condition, Pseudomonas aeruginosa and Pseudomonas putida, 96‐hour incubation.	27.9 % PFOA and 47.3 % PFOS (RE) by *Pseudomonas aeruginosa*, 19.0 % PFOA and 46.9 % PFOS (RE) by *Pseudomonas putida*	^[91]^
	PFOA	Anaerobic *Acidimicrobium* sp. strain A6, with PAA‐coated ferrihydrite, 40 days incubation.	Unspecified	^[92]^
	PFOA	Biosolids with A*cidimicrobiaceae* sp. strain A6 and ferrihydrite, Feammox enrichment culture, anoxic condition, 150 days incubation.	Unspecified	^[93]^
	6 : 2 FTOH, TFMAA	Landfill soil, mixed species, aerobic microbial culture, room temperature, pH 7.0, 32 days.	~28 % (DE)	^[94]^

#### 2.1.3 Thermal Treatment Techniques

The wide range of thermal degradation techniques for PFAS include pyrolysis, combustion, incineration, smouldering combustion, gasification, hydrothermal liquefaction and microwave hydrothermal treatment;[^18^, ^71^] known to mineralize PFAS compounds under certain specified conditions effectively. For instance, the thermal degradation of PFOS involves a 3‐step random chain scission pathway, starting with C−S bond cleavage, then defluorination and then radical chain propagation reactions.^[72]^ The most efficient calcium additive for PFAS defluorination was Ca(OH)_2_ because the hydro‐defluorination process can change the C−F bonds in PFAS into C−H bonds. Generally, PFAS degradation mechanisms involve disrupting C−C and C−F bonds, with H/F exchange and chain shortening being predominant pathways.^[73]^ With the following sequence of the counterions: NH_4_^+^<Cs^+^<K^+^<Ag^+^<Pb^2+^<Na^+^<Ca^2+^=Ba^2+^<Li^+^ the thermal stability of PFOA salts increased.^[74]^ Under the appropriate thermal conditions, PFOA and PFOS may be rapidly broken down and mineralized. Inorganic calcium, such as calcium hydroxide (Ca(OH)_2_), calcium oxide (CaO), and calcium carbonate (CaCO_3_), often aid in this process and also in fixing CF_4_ and C_2_F_6_ greenhouse gases.[^18^, ^75^] Low temperature (300–900 °C) PFAS destruction, simultaneous fluorine capture and mineralization to CaF_2_ were observed by Riedel and coworkers.^[76]^ As the amount of carbons in PFAS increases, the temperature needed to decompose them lowers; calcium additives also lower the PFAS decomposition temperature.[^74^, ^75^] During PFAS decomposition, the corrosive gas hydrofluoric acid (HF) formed could be neutralized with a base such as milk of lime, NaOH or KOH.^[77]^ CaO and Ca(OH)_2_ exhibit higher defluorination capabilities than CaCO_3_.^[78]^ While CaO delivers high mineralization efficiency, Ca(OH)_2_ promotes defluorination at temperatures <400 °C. CaO crystals splitting from heating supplies fresh surfaces for PFAS reaction.^[75]^ Overall, fluorine mineralization by calcium additives is a promising strategy for PFAS treatment.^[73]^ The effectiveness of any method is often evaluated based on degradation efficiency, defluorination efficiency, and degradation rate.^[70]^ One of the best possible remediation technologies could be thermal treatment, which can eradicate HF emissions and destroy PFAS in any short‐chain forms from the environment.

### 2.2 Chlorinated Polymers

PVC is a versatile chlorinated polymer widely used in various applications due to its durability and flexibility as discussed above. The PVC waste landfilling and ocean dumping have aroused concerns about its long‐term impact on our ecosystem and human health, and hence, extensive research is underway for a sustainable solution for PVC degradation. The first ever proposal of PVC degradation mechanism and thermal degradation studies started in the 1950s[^95^, ^96^] as shown in Figure 1. The degradation of PVC is a multi‐stage process that includes HCl elimination, formation of conjugated polyenes, plasticizer migration, and changes in molecular weight due to chain scission and/or cross‐linking.^[97]^ The HCl release could be because of the lower average BDE of the C−Cl (356.4 kJ/mol) than C−C (375.0 kJ/mol).^[98]^ These processes result in visible discoloration, stickiness, and cracking of the PVC materials; long‐term exposure to environmental factors such as heat, water, sunlight, and various chemical compounds leads to irreversible structural changes and a decrease in molecular weight.^[99]^ The recent chemical, biological and physical PVC degradation approaches are discussed herein as shown in Figure 4 along with their de‐chlorination/ de‐hydrochlorination efficiency.

#### 2.2.1 Chemical Degradation Techniques

Recent studies have focused on various methods to address environmental concerns and recycling challenges. Chemical recycling technologies, such as wet treatments and de‐chlorination processes, have shown promise in transforming PVC waste into valuable chemicals and fuels.^[100]^ However, the effectiveness of these techniques varies depending on the polymer composition and environmental conditions. Thiophenol in the presence of K_2_CO_3_ and N,N‐diisopropylethylamine were successful in reaching a maximum substitution ratio of up to 35.7 % to remove the Cl in PVC, for converting waste PVC into value‐added product feedstock.^[101]^ De‐chlorination of waste flexible PVC rich in di‐(2‐ethylhexyl) phthalate reached 98.7 % at 300 °C by using low‐temperature critical aqueous ammonia.^[102]^ Another study by Xiu and coworkers^[103]^ demonstrated an alkaline system more conducive to de‐chlorinate DEHP plasticized PVC as the de‐chlorination efficiency of the subcritical water‐C_2_H_5_OH system at 250 °C was only 75.33 %, while that of the subcritical water‐NaOH system exceeded 90 %; and was close to 100 % at 350 °C which could be ascribed to the neutralization‐induced trapping action of alkaline NaOH on the acidic de‐chlorination product HCl. De‐chlorination efficiency exceeded 90 % at 250°C by using the near‐critical methanol (NCM) method for the medical waste PVC treatment at the optimal reaction temperature, solid‐to‐liquid ratio, and reaction time.^[104]^ Another study by Qi and coworkers^[105]^ uncovered that the partial oxidation treatment process in subcritical water for waste PVC could reach 95 % de‐chlorination efficiency at 300°C. The PVC pressurized hydrolysis in an autoclave reactor reached 95 % de‐chlorination efficiency under an optimal reaction time of 2 hours and 240 °C.^[106]^ Electrocatalysis techniques to de‐halogenate compounds with chlorines over Pd−In/Al_2_O_3_ catalyst and also to generate highly efficient hydroxyl radicals to degrade organic pollutants have already been studied previously.[^107^, ^108^, ^109^] An electro‐Fenton‐like method was further developed using a TiO_2_/graphite cathode to degrade PVC by Miao and coworkers^[110]^ indicating that the oxidative degradation and PVC de‐chlorination occurred by removing approximately 56 wt.% of PVC and exhibited 75 % de‐chlorination efficiency as tabulated in Table 2. During electro‐catalysis, hydroxyl radicals initiate oxidative attack on the saturated PVC altering them to smaller organic compounds and further oxidation to gaseous molecules. Direct photolysis with UV‐activated persulfate (PS) oxidation at neutral pH for 35 hrs accelerated the de‐chlorination efficiency with higher initial PS concentration as discussed in Table 2.

**Table 2:** Various PVC treatment studies recently (within 5 years).

Methods	Catalyst/ electrode/ substrate used	Treatment conditions	Treatment highlights	References
Photodegradation	ZnO/UiO66‐NH_2_ heterojunction photocatalyst	300 W Xe lamp, 21 A, 14 V, 25.4 mm bulb window, sealed quartz reactor with air at 25 °C.	Efficient H_2_ production coupled with improved acetic acid production	^[113]^
	Graphite carbon nitride (g‐C_3_N_4_)	HT‐XD‐150 L xenon lamp, 55 mW/cm^2^ irradiation, 38 °C, 120 hrs.	De‐chlorination coupled with CO_2_ and H_2_O production	^[146]^
	Direct photolysis	Low‐pressure Hg lamp, UV‐activated persulfate (PS) oxidation, 7 pH, 35 hrs.	Accelerated de‐chlorination efficiency with higher initial PS concentration	^[111]^
	Nb_2_O_5_ atomic layers	300 W Xe lamp with a standard AM 1.5G filter, ambient temp. and pressure.	High‐energy‐density C_2_ fuels	^[147]^
	Nano‐graphite coupled with TiO_2_ photocatalyst	300 W medium‐pressure UV at 365 nm, ambient conditions, 30 hrs.	Conversion of PVC composite film to CO_2_	^[114]^
Electrochemical	CeO_2_ doped PbO_2_ composite anode	Batch process, Na_2_SO_4_ electrolyte, 800 rpm, current density (10–60 mA/cm_2_), 6 hrs.	PVC weight loss rate was 38.67±1.91 %	^[148]^
	Both graphite electrodes	Galvanostatic electrolysis under constant current 10 mA, NBu_4_BF_4_ electrolyte, 400 rpm, 16 hrs.	PVC de‐chlorination (23.3 %) coupled with arene chlorination	^[149]^
	TiO_2_/graphite cathode	Batch process, Na_2_SO_4_ electrolyte, 800 rpm, 6 hrs, O_2_‐saturated optimal conditions.	De‐chlorination efficiency (75 %)	^[110]^
Thermal	PbO catalyst	Co‐pyrolysis, horizontal quartz tube reactor, 100 ml/min N_2_ flow, 300–600 °C, 10 °C min^−1^.	45.57 % HCl capture	^[31]^
	Ru/Al_2_O_3_ catalyst	Stainless‐steel autoclave reactor, hydrogenolysis, 180 °C, 600 rpm, H_2_ pressure (2 MPa).	Cl utilization efficiency >80 % (using tetrahydrofuran as an acceptor)	^[150]^
	Rhodium catalyst	Tandem dehydrochlorination–hydrogenation, 20 bar H_2_, 750 rpm, 100–180 °C.	81 % dehydrochlorination	^[151]^
	(Xantphos)RhCl catalyst	H_2_ donors, 5 ml solvent, 110 °C, 2 days.	Complete de‐chlorination of PVC	^[152]^
	EAFD	Co‐pyrolysis, U‐shape reactor under N_2_ flow, 30 mins, 10 °C.min^−1^.	Zn recovery (~100 %), Pb (~76 %), and Fe (~64 %) capturing HCl	^[130]^
	Franklinite (ZnFe_2_O_4_)	Co‐pyrolysis, quartz tube, 30 mins, 10 °C.min^−1^.	T_onset_ reduced to 235 °C, complete franklinite chlorination utilizing HCl	^[140]^
	EAFD	Oxidative treatment, P_O2_=21 kPa, U‐shape reactor, 30 mins, 10 °C.min^−1^.	Zn and Pb recovery at ~100 % capturing HCl	^[142]^
	ZnO (or) KOH as Cl‐fixative	Tube furnace, 260 °C at 5 °C min^−1^, 30 min, 100 ml/min N_2_ flow.	84.48 % and 94.15 % Cl fixation by ZnO and KOH resp.	^[134]^
Biodegradation	*Coleoptera Tenebrionidae* in *Tenebrio molitor* larvae gut	CFBA medium, 30 °C incubation, 30 days, 180 rpm.	6.13 % DE	^[153]^
	*Citrobacter koseri* in *Zophobas atratus* larvae gut	NB medium, 37 °C incubation, 30 days, 200 rpm for 5–6 hrs.	2.06 % DE	^[154]^
	*Bacillus licheniformis*, *Achromobacter xylosoxidans* (Bacterium) *Aspergillus niger* and *Aspergillus glaucus* (Fungi)	Incubation at 25, 37 and 45 °C, 4 weeks.	17 % (by bacterium) and 32 % (by fungi) DE	^[155]^
	*Klebsiella* sp. EMBL‐1 in *Spodoptera frugiperda* larvae gut	MSM liquid medium, 30 °C incubation, 90 days, catalase‐peroxidase enzyme.	19.57 % DE	^[116]^
	*Vibrio* sp., *Alteromonas australica* and *Cobetia* sp. (marine bacterial isolates)	BH media, 150 rpm, 30 °C incubation, 60 days.	1.76 % DE	^[156]^

**Figure 6:**
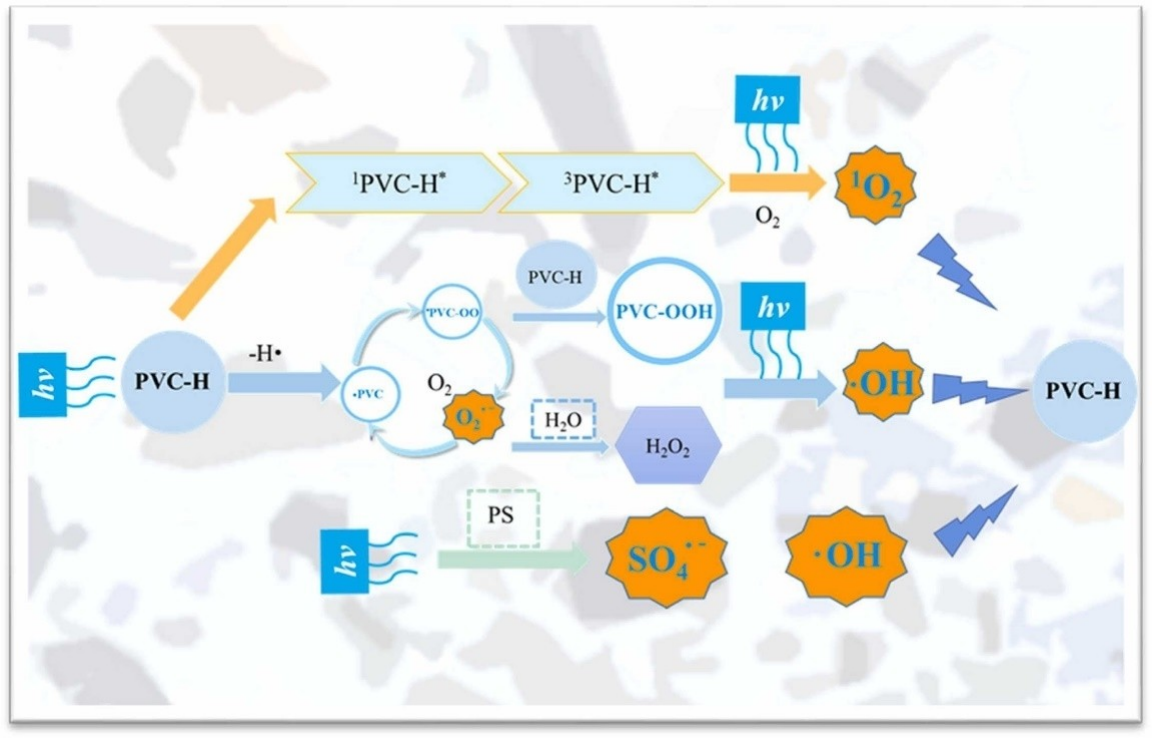
Direct photolysis pathway for PVC treated by UV‐activated PS system. Reproduced with permission.^[111]^ Copyright © 2022, Elsevier Ltd.

The proposed mechanism for the same by Ouyang and coworkers^[111]^ is shown in Figure 6. During the photocatalysis by UV, the irradiation is endowed with enough energy to cleave initially the C−C bond or C−H bond, and de‐chlorination (C−Cl bond) was the first step in the aging of PVC.^[112]^ The synergistic effect of ZnO/UiO66‐NH_2_ heterojunction exhibited an exciting activity for the photocatalytic valorization of polylactic acid (PLA) and PVC into acetic acid, coupled with the H_2_ production.^[113]^ Zhang and coworkers^[114]^ have also shown promising photocatalytic degradation of PVC composite films by co‐doping nano‐graphite and TiO_2_. Recently, Yan and coworkers^[115]^ demonstrated catalytic ozonation of polyvinyl alcohol using copper‐manganese/γ‐alumina catalysts and proved effective in breaking down long polymer chains, achieving up to 99.3 % removal in optimal conditions, which could be experimented with PVC. However, all these techniques end up in the production of corrosive HCl gas and no chemical techniques arrest these free chlorine radicals for restricting their atmospheric release.

#### 2.2.2 Biological Degradation Techniques

The microbial degradation of PVC could emerge as an eco‐friendly solution to a plastic waste management facility. Various bacterial strains capable of degrading PVC have been isolated from environmental samples and insect guts using enrichment culture techniques. These microorganisms, including *Micrococcus* sp. and *Klebsiella* sp. EMBL‐1, can utilize the PVC as a sole carbon source and feed on it, demonstrating its ability in depolymerizing as well as metabolizing it.[^116^, ^117^] The biodegradation process involves the release of chloride, CO_2_ production, and increased cell density. Enzymes such as catalase‐peroxidase, dehalogenases, and oxygenases are potentially involved in PVC degradation.^[116]^ The microbial strains such as the *Bacillus* sp. AIIW2 (marine strain), *Psedomonas* sp., *Brevibacterium* sp. and fungal strains such as *Curvularia* sp., *Trogia buccinalis* and *Phanerochaete chrysosporium* offer a promising strategy for PVC degradation and could potentially lead to the conversion of waste PVC into high‐value chemicals.[^118^, ^119^, ^120^, ^121^] Many recent studies have explored the enzymatic degradation techniques for PVC and other synthetic plastics, including manganese peroxidase, laccases, and catalase‐peroxidase, showing its potential in PVC degradation.[^116^, ^122^, ^123^] The enzyme manganese peroxidase demonstrated the highest binding affinity of −2.7 kcal/mol for the PVC among several other tested enzymes.^[122]^ Similarly, fungal laccases and peroxidases have also shown exciting results in degrading PVC.^[123]^ A bacterial strain isolated from an insect larvae gut (*Klebsiella* sp. EMBL‐1) as tabulated in Table 2 was found to be capable of depolymerizing and utilizing the PVC as its sole energy source.^[116]^ Typically, the enzymatic degradation process involves two stages: (i) the adsorption of enzymes on the polymer surface, followed by (ii) the hydro‐peroxidation or hydrolysis of the bonds.^[124]^ These findings suggest that enzymatic degradation techniques could offer potential solutions for addressing waste PVC pollution in the future, though it is an arduous and time‐consuming process.

#### 2.2.3 Thermal Degradation Techniques

Several PVC thermal treatment processes have already been studied to obtain a high liquid oil yield consisting of desirable arenes and also co‐generate syngas. Recently, a ternary molten carbonate salt mixture (Li_2_CO_3_‐Na_2_CO_3_‐K_2_CO_3_) gasification technique exhibited promising results, with gasification efficiencies up to 41.2 % at 750 °C and high chlorine retention, while also reducing PCDD/F generation at <19 pg/g PVC.^[125]^ Pure PVC pyrolysis is anticipated to yield high‐value pyrolytic oil that consists of polycyclic aromatic hydrocarbons (PAHs) acclaimed via the release of two to four aromatic rings after a 3‐stage degradation mechanism that includes inner cyclization and aromatic chain scission in addition to the de‐chlorination.^[126]^ However, catalytic pyrolysis of the waste PVC is being widely studied globally to address environmental concerns and also to improve its recycling efficacy. Multiple catalysts including MOs and zeolites, are also being examined for their de‐chlorination effects and product distribution. Pure PVC exhibits a couple of thermal degradation phases apparently, viz. the de‐hydrochlorination phase wherein HCl egression is marked as the first stage (230–380 °C) and in the second stage (390–550 °C) charring occurs from the thermal cracking of the prevailing hydrocarbons.^[127]^ During co‐pyrolysis, the presence of additives such as ZnO decreased the degradation onset temperature (T_onset_) by 58 °C^[128]^ whereas Ca(OH)_2_ delayed the T_onset_ to 270 °C.^[127]^ Likewise, Cheng and Liang^[129]^ found that the addition of metal chlorides like ZnCl_2_ and BaCl_2_ can decrease the dehydrochlorination temperature to about 200 °C during the co‐pyrolysis. On the contrary, Al‐Harahsheh and coworkers^[130]^ found that the presence of NaCl and KCl from electric‐arc furnace dust (EAFD) delayed the PVC dehydrochlorination by 20 °C. The ZSM‐5 catalyst was found to promote HCl release and reduce organochloride content in pyrolysis oil.^[131]^ Among the different zeolites studied, MCM‐41 showed higher ex‐situ catalytic cracking activity in PVC degradation, lowering the conversion temperature and reducing the formation of PAHs; recommending a two‐step process involving non‐catalytic de‐chlorination at around 300 °C followed by catalytic degradation with MCM‐41 at higher temperatures for optimal results.^[132]^ The recent co‐pyrolytic study of PVC with PbO by Kuttiyathil and coworkers^[31]^ revealed a 45.57 % HCl capture. During the co‐pyrolysis of PVC microplastics with FeAlO_x_ catalyst, the C−Cl bond cleavage released more HCl, resulting in fewer chlorinated compounds in the target value‐added products.^[133]^ The one‐pot reactions of ZnO and KOH with PVC by Zhou and coworkers^[134]^ were successful in the de‐chlorination carbonization attempt by fixing the chlorides into Zn_2_OCl_2_ ⋅ 2H_2_O and KCl respectively as shown in Table 2. Hu and coworkers^[131]^ found that CaO and Fe_2_O_3_ mainly acted as an adsorbent for fixing HCl without a significant cracking effect positively correlated to alkalinity and these metal oxide catalysts slightly lowered the initial temperature of PVC degradation. The in‐situ removal of chlorine during co‐pyrolysis of PVC with Pingshuo coal in a two‐stage fixed‐bed reactor was studied by Wang and coworkers^[135]^ wherein the CaO bed was placed in the second stage of the reactor. The synergistic effect of PVC with Pingshuo coal had a positive effect on the tar yield (with lower chlorine content) and trimmed down the HCl yield due to the presence of the CaO bed; the dehydrochlorination efficiency of CaO reaching 78.1 % at 700 °C. The presence of calcium additives during the pyrolysis of commercial PVC plastics promoted heavy constituent cracking in the volatile phase favouring more light fractions in the tar accompanied by the chlorine fixation effect of the calcium additives.^[136]^ In the HCl absorption study by limestone in hot flue gases, either calcium hydroxy‐chloride or an oxide form of calcium is the first product formed leading to the end product formation of CaCl_2_; which was true for the reactions of both calcined and uncalcined limestone at 650 and 850 °C.^[137]^ These calcium additives′ HCl‐capturing phenomenon could be exploited in any thermal treatment of PVC, endorsing the non‐release of corrosive HCl gas into the atmosphere. A recent thermodynamic simulation suggests that zinc and manganese present in spent alkaline batteries (SAB) can be extracted via pyrochar leaching upon the co‐pyrolysis with PVC, due to the excellent chlorine fixation by zinc oxide and manganese oxide present in SAB via the dehydrochlorination route.^[138]^ EAFD has been considered one of the best dechlorination agent due to its rich mineralogical composition such as franklinite (ZnFe_2_O_4_), zincite (ZnO), CaO, dicalcium ferrite (Ca_2_Fe_2_O_5_), Ca(OH)_2_, periclase (MgO), pyrolusite (MnO_2_), PbO, quartz, iron chromite (Fe,Cr)_2_O_3_, magnetite (Fe_3_O_4_), hematite (Fe_2_O_3_), wüstite (FeO), siderite (FeCO_3_) and halite (NaCl).^[139]^ It is potent enough to capture the HCl release during co‐pyrolysis[^130^, ^140^] and even at oxidative conditions.[^141^, ^142^] The obtained pyrochar when subjected to subsequent water leaching, was able to recover both zinc and lead (100 %)[^142^, ^143^] with some exception to iron, endorsed by thermodynamic assessment as well. Microwave‐assisted heating studies of EAFD^[144]^ with PVC using separated electric and magnetic fields also resulted in 97 % recovery of Zn^[145]^ via subsequent leaching. The recent PVC degradation studies tabulated in Table 2, which contains the investigations spanning the last five years, endorse that any controlled thermal degradation technique could serve as a viable solution in mitigating the associated risk when a suitable metal oxide additive is exploited.

### 2.3 Brominated Polymers

Though BFRs have significantly improved the fire safety concern in various consumer products, anxieties have arisen regarding their potential environmental and health impacts after their usage. These compounds are toxic, persistent in nature and bio‐accumulative, hence leading to their biomagnification in the environment and human tissues. To address these issues, the development of an effective degradation technique that targets BFRs to break down into less harmful substances or even to immobilize them has become increasingly important. In 2010, the degradation pathway studies for TBBPA were initially done by Nyholm and coworkers^[157]^ as shown in Figure 1. The challenge of degrading BFRs lies in their stable chemical structure, which resists their natural breakdown processes. As discussed earlier, the traditional intuitive disposal methods such as incineration and landfilling could lead to the release of harmful by‐products including HBr and PBDD/Fs or contaminant leaching into the aquifers beneath.^[33]^ These toxic gas emissions could be attributed to the significantly weaker bond energies as that of the aromatic C−Br BDE (337.4 kJ/mol) being weaker than the C−H BDE (412.5 kJ/mol).^[158]^ Therefore, innovative degradation techniques to mitigate the environmental impact of BFRs are required to ensure safer waste management practices. Herein, recent degradation strategies such as natural substance catalysis, new Fenton‐like catalysis, and electrocatalytic degradation^[159]^ including physical, chemical, and biological methods as shown in Figure 4, are being explored to discuss their potential for mitigating the risks associated with BFRs; thermal catalytic approach showing promise due to its rapidness and effectiveness.

#### 2.3.1 Chemical Degradation Techniques

**Figure 7:**
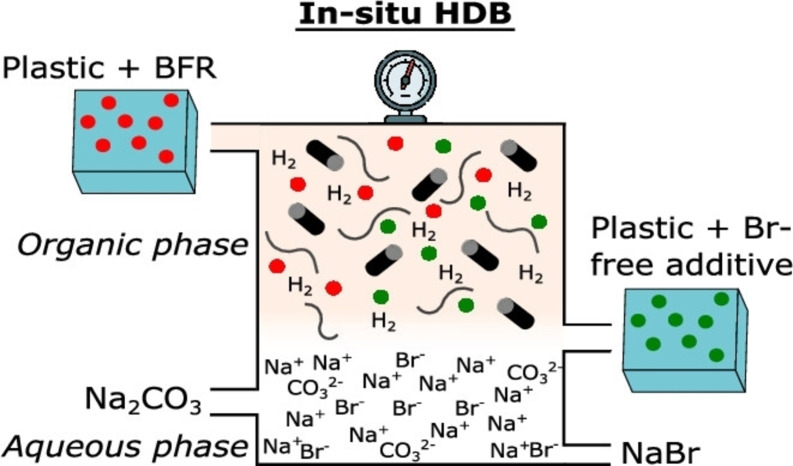
In‐situ catalytic debromination scheme has been reproduced with permission.^[162]^ Copyright © 2024, American Chemical Society.

Developing an effective and promising chemical degradation technique for the BFRs is the research focus these days. Catalytic degradation methods, including photocatalytic and nano‐catalytic reduction, have already shown promising approaches. TBBPA has been detected in multiple environmental compartments and could be treated through adsorption, ozonation, and anaerobic degradation.^[160]^ The solvothermal technique has also shown some capacity in removing BFRs from electrical and electronic waste plastics, with methanol proving to be an optimal solvent for TBBPA removal under certain specific conditions.^[161]^ An efficient catalytic hydro‐debromination (HDB) reaction of conventional BFRs without any pretreatment, using Pd/C catalyst under 75–100 °C and 5–10 bar H_2_ to yield up to 99 % industrially valuable products within 6–24 hrs of reaction time in a 3 mL homemade autoclave as shown in Figure 7.^[162]^ Mechanochemical debromination of HBCD with the aid of SiO_2_/Al‐based additives conducted in a planetary ball mill demonstrated effective degradation.^[163]^ Similar mechanochemical debromination of a novel BFR, allyl 2,4,6‐tribromophenyl ether (ATE) entailed the highest debromination efficiency of 23 % with the Fe/Al_2_O_3_ catalyst.^[164]^ In a recent study by Dar and coworkers^[165]^, a strong reactive oxidant Fe(VI) in an acidic medium can efficiently degrade many bromophenols in water; the removal efficiency being directly proportional to the oxidant dose. Gripon and coworkers^[166]^ extracted BFRs from real e‐waste using supercritical carbon dioxide (*sc*‐CO_2_); the maximum extracted bromine being 43.5±0.9 % improves the extraction rate by adding ethanol as a co‐solvent. Highly saline industrial wastewater was exploited with the reactive chlorine species due to the low redox potentials along with co‐existing hydroxyl radicals (⋅OH) during the electrochemical oxidation process using electroactive membrane filtration (EMF) for treating tetrabromobisphenol S (TBBPS). It achieved a high removal efficiency of 99.1±0.5 %.^[167]^ The fluorine‐doped titanium suboxide is an electrochemically reactive membrane via a more efficient outer‐sphere reaction and the hydroxylation route facilitated the electrochemical conversion of TBBPA and its derivatives.^[168]^ Copper oxide nanoparticles have shown potential as catalysts for BFR degradation, with proposed mechanisms involving highly reactive hydroxyl and superoxide radicals generation.^[169]^ Therefore, the development of an appropriate electrode material is crucial for DE improvement, with recent advances being focused on new electrocatalysts for brominated organic pollutant degradation.^[170]^ Recent research on nano zero‐valent iron (nZVI) techniques for BFRs has also shown promising results; exhibiting higher reactivity and cost‐effectiveness when compared to micro‐ZVI while treating various brominated contaminants. Comparisons of different nZVI‐based nanoparticles revealed that SiO_2_@FeOOH@Fe and nano‐Ni/Fe bimetallic particles have higher reusability and stability, with SiO_2_@FeOOH@Fe effectively promoting the PBDEs remediation exclusively by preventing the iron leaching.^[171]^ Bimetallic nanoparticles, particularly nZVI/Pd, have shown enhanced dehalogenation kinetics for mono‐ to tri‐BDEs compared to nZVI alone. However, when impregnated in activated carbon, the debromination process was slower due to the factors such as heterogeneous distribution and immobilization of contaminants.^[172]^ Excitingly, a submicron zero‐valent iron (smZVI) coated with FeC_2_O_4_ ⋅ 2H_2_O layers (OX‐smZVI) is emerging as a replacement for nZVI in PBDEs remediation. Interestingly, OX‐smZVI exhibited a better removal efficiency with OX/Fe=8 % mol/mol towards BDE‐209 with 99.72 % over nZVI and smZVI.^[173]^ For the various other PBDE congeners studied, photocatalysis is the most widely reported remediation method for BDE‐47 and BDE‐209 with debromination efficiency reaching 90 % over reduced graphene oxide loaded TiO_2_ (RGO/TiO_2_) photocatalyst.[^174^, ^175^] Green‐synthesized NiO@CuHCF nanohybrids demonstrated a debromination efficiency of 75.4‐82.3 % during the photodegradation of TBBPA and ATE via natural sunlight for two hours (12–2 pm) as shown in Table 3.^[176]^ The novel BFRs such as penta‐bromo‐toluene (PBT) and hexa‐bromo‐benzene (HBB) were subjected to photocatalytic oxidation and reduction processes using sulphate radical modified carbon nitride (SO_4_^2−^@CN) catalyst under visible light irradiation and a removal efficiency of 100 % was observed.^[177]^ The direct irradiation of UV light upon any legacy/novel BFRs was found to exhibit appreciable debromination efficiency as tabulated in Table 3.[^29^, ^178^] Moreover, TiO_2_ photocatalysis could be identified as the most suitable for treating BFR contamination in waters, as it achieved a higher debromination and mineralization degrees. Photodegradation kinetics of highly brominated entities such as TDBP‐TAZTO and DBDPE, are influenced by factors including UV wavelength, intensity, and also the solvent type.^[179]^ These degradation processes could probably result in a variety of breakdown products, potentially complicating the ecological risk assessments. As shown in Figure 1, a comprehensive study by Peng and coworkers^[180]^ utilizing the UV/TiO_2_/H_2_O_2_ process targeting the photocatalytic degradation of TBBPA exhibited an impressive DE recently. In a nutshell, photochemical degradation techniques have the potential in BFR mitigation, but further research is needed to address the formation of degradation products and optimization of the treatment conditions.

**Table 3:** Recent BFR degradation studies (within 5 years) by various methods.

Methods	Catalyst/ electrode/ substrate used	Treatment conditions	Treatment highlights	References
Photodegradation	Green‐synthesized NiO@CuHCF nanohybrids	TBBPA and ATE, pH (3−11), sunshine intensity (160–815±5 lux), 2 hrs, room temperature.	75.4‐82.3 % debromination efficiency (DB)	^[176]^
	Direct irradiation	DBDE, TTBPT and HBCD, Xenon lamp LC8, 19 mW/cm^2^, 365 nm.	>50 % (DB)	^[29]^
	Direct irradiation	DBDE, LC8 Xenon light source, UV‐visible light, <20 mbars, 15 mins.	88‐97 % (DB)	^[178]^
	Direct irradiation	TDBP‐TAZTO and DBDPE, UV lights at 310 −350 nm, 1.8 mw/cm^2^ intensity.	Degradation half‐lives (t_1/2_) of 91.2 days (during natural sunlight)	^[179]^
	Singlet oxygen (^1^O_2_) excitation from humic acid	TBBPA, LED white light (λ >400 nm, 5 W), room temp., 45 mins.	100 % (DE)	^[210]^
Electrochemical	Reactive chlorine species	TBBPS, electroactive membrane filtration, NaCl (or) Na_2_SO_4_ as electrolyte, 500 rpm, 30–150 L.m^−2^.h^−1^ flux, 1.45 kWh.m^−3^.	99.1 ± 0.5 % (RE)	^[167]^
	Metal‐free graphene oxide	TBBPA, 150 rpm, pH 5.5, dark and ambient condition, 4 hrs, Na_2_SO_4_ electrolyte.	Complete TBBPA degradation	^[211]^
	Ti/SnO_2_‐Sb/PbO_2_‐Ce anode	TBBPA, Electrochemical filtration reactor, alkaline electrolyte, 0.11 kWh/m^3^, 1–4 V.	100 % (RE)	^[212]^
	Nano Pd/Ni foam electrode	TBBPA, 100 mM Na_2_SO_4_ electrolyte, −1.2 V, room temp., 3 hrs.	99.7 % (DE)	^[213]^
	F‐doped TiSO anode	TBBPA, 7.8±0.24 mA cm^−2^, 4–1628 LMH, NaClO_4_ electrolyte, room temp.	99.7 % (RE)	^[168]^
Thermal	Zn‐based additives	TBBPA‐DBPE and TBBPA‐DAE, co‐pyrolysis in a horizontal quartz tube reactor, 100 ml/min N_2_ flow, 300–500 °C, 10 °C min^−1^.	HBr capture >92 %	^[190]^
	PbO	TBBPA‐DAE, co‐pyrolysis in a horizontal quartz tube reactor, 100 ml/min N_2_ flow, 300–600 °C, 10 °C min^−1^.	HBr capture >80.04 %	^[31]^
	Ca(OH)_2_	TBBPA‐DAE, ATE and TBP, co‐pyrolysis in a horizontal quartz tube reactor, 100 ml/min N_2_ flow, 300–500 °C, 10 °C min^−1^.	80.49 % (DB)	^[204]^
	Ca(OH)_2_	TBBPA, horizontal quartz tube reactor, 100 ml/min N_2_ flow, 300–500 °C, 10 °C min^−1^.	100 % (DB)	^[201]^
	Fe_2_O_3_	TBP, co‐pyrolysis in a horizontal quartz tube reactor, 100 ml/min N_2_ flow, 300–500 °C, 10 °C min^−1^.	87 % (DB)	^[195]^
Biodegradation	*Dehalococcoides mccartyi* CG1 strain	*β‐*TBCO and DPTE, bicarbonate‐buffered mineral medium, 30 °C incubation in dark, no shaking, 120 hrs.	Br isotope fractionation	^[214]^
	*Rhodopseudomonas palustris*	HBCD, batch, PNSB medium, 35 °C, dark, aerobic, 72 hrs incubation.	40 % (DE)	^[215]^
	*Dehalococcoides mccartyi* CG1 strain	TBBPA, DCB‐1 medium, dark incubation, 30 °C, no shaking, anaerobic, 10 days.	Complete debromination of TBBPA to BPA	^[216]^
	*Clostridium* spp. and *Bacillus* spp.	HBCD, soil suspension, 35 °C, dark, aerobic, 150 rpm, 16 days incubation.	77.9 % (RE)	^[217]^
	*Rhodopseudomonas palustris* YSC3 strain	HBCD, batch experiments, 150 rpm, PNSB medium, 35 °C, 10 days incubation.	>20 % (DE)	^[218]^

#### 2.3.2 Biological Degradation Techniques

Various microbial degradation techniques are being studied for BFRs, PBDEs in particular. In both anaerobic and aerobic microbial degradation processes, functional genes were found to play crucial roles in the degradation pathways. Biotechnological approaches such as synthetic microbial consortia and genetic engineering techniques, have also been explored to enhance plastic bioremediation and degradation.^[181]^ The microbial degradation of PBDEs is particularly important due to their persistence in the environment and potential health risks. Five enzymes encoded by *tbpA*, *tbpB*, *tbpC*, *tbpD* and *tbpE* in the genome of strain GZT possessed an extraordinary ability to simultaneously de‐brominate cum mineralize the novel BFR, 2,4,6 tri‐bromophenol (TBP); cytochrome P450 reductase encoded by *tbpA* gene possessing removal efficiency of 98.8 %.^[182]^ Interestingly, a coupled system consisting of microorganisms with reductive dehalogenase genes like *Dehalococcoides*, *Dehalobacter*, *Dehalogenimonas* and carboxymethyl cellulose (CMC‐nZVI) showed the best degradation effect reaching approximately 90 % within a span of 30 days.^[183]^ The synergistic effect in the removal of heavy metals combined with BDE‐209 (belonging to the PBDE group) by *Microbacterium* Y2 (MY2) and sulfurized nZVI co‐incubation system accounted for an appreciable 78.8–88.4 % DE in the presence of Cr(VI); whereas 95 % removal in the MY2@S‐nZVI/Ni(II) system as highly reactive Ni^0^/Fe^0^ bimetals were quickly formed by the reduction of Ni(II) by sulfurized nZVI.^[184]^ Future research should focus on developing eco‐friendly, cost‐effective, and pragmatic catalytic techniques for BFR degradation, thereby improving the understanding of intriguing microbial degradation mechanisms.

#### 2.3.3 Thermal Degradation Techniques

**Figure 8:**
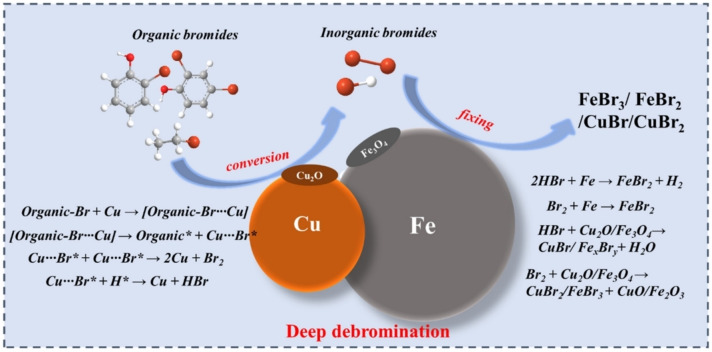
Debromination mechanism during the co‐pyrolysis of Cu (or) Fe with waste‐printed circuit boards. Reproduced with permission.^[189]^ Copyright © 2024, MDPI licensed under CC BY 4.0.

Recent research has focused on developing novel catalytic techniques for the thermal degradation of BFRs in the environment. In 2003, thermal degradation studies on brominated acrylonitrile‐butadiene‐styrene copolymer and brominated high‐impact polystyrene were done as shown in Figure 1.[^185^, ^186^] Thermal decomposition of BFRs typically occurs at temperatures between 280–900 °C, with both condensed and gas phase reactions contributing to the process; the prime target is the restriction of HBr and PBDD/Fs exploiting the metal oxides. MOs are preferred for the catalytic debromination of BFRs due to their Lewis acid/base cum redox properties, thermal stability, reaction activation energy (E_a_) reduction and thereby reducing the energy consumption for pyrolysis.^[187]^ The potential of hematite (Fe_2_O_3_) as a debromination agent for TBP was studied by Mousa and coworkers^[188]^ and found that the TBP conversion reached ~33 % while reducing HBr emission by 45 %. Copper–iron (Cu/Fe) bimetal exhibited a maximum debromination efficiency of 97.14 % at 600 °C during the co‐pyrolysis with waste‐printed circuit boards containing BFRs wherein the inorganic bromine was fixed either by Cu (or) Fe in the pyrolytic residue.^[189]^ The proposed debromination mechanism is illustrated in Figure 8, fixing the HBr by forming FeBr_2_ and CuBr_2_. The toxic HBr capture approaching >92 % was achieved with ZnO treatment upon co‐pyrolysis of a novel BFR, tetrabromobisphenol A diallyl ether^[190]^ and a patent has been obtained for the same (US Patent 12,202,780 B1).^[191]^ Similarly, tetrabromobisphenol A diallyl ether was co‐pyrolyzed with PbO to achieve an HBr capture >80.04 %.^[31]^ Fe/Al_2_O_3_ exhibited a debromination efficiency of ∼75 % promoting phenol production and eliminating brominated compounds from pyrolytic oil, followed by Fe/MgO at ∼60 % and MgO at ∼55 % during the co‐pyrolysis of polymeric blends that consist of polypropylene, high‐impact polystyrene, acrylonitrile‐butadiene‐styrene and polycarbonate in the presence of TBBPA.^[192]^ The computer casing plastics waste containing BFRs when subjected to a two‐stage catalytic pyrolysis reactor using iron and nickel bimetallic Fe/Ni modified MCM‐41 catalysts was found to reach higher debromination efficiency for catalysts with more Fe loading.^[193]^ The latent capacity of alumina (Al_2_O_3_) as a debromination agent via HBr fixation during the co‐pyrolytic handling of e‐waste was reported by Ali and coworkers.^[194]^ The oxidative and pyrolytic disintegration of a gaseous stream of TBP over Fe_2_O_3_ in a two‐stage catalytic pyrolysis reactor; combined with the prediction of thermogravimetric data using Machine Learning (ML) techniques also endorsed the bromine fixation as FeBr_2_ in the pyrolytic char by a significant reduction of HBr emission from 48.7 to 6.2 mg/g.[^195^, ^196^, ^197^] EAFD has also exhibited appreciable debromination properties upon co‐pyrolysis with TBBPA by capturing the toxic HBr emanated. The subsequent water leaching of the pyrochar (containing several metal bromides) could lead to a maximum possible recovery of 100 % of Pb, ~91 % zinc and<30 % iron depending upon the leaching solution pH^[139]^ endorsed by the thermodynamic analysis using FactSage^[198]^ and degradation kinetics studies.^[199]^ Whereas the EAFD‐TBBPA mixture when subjected to microwave treatment led to a lower metal recovery^[200]^ during subsequent leaching of the pyrochar. However, a complete and deep removal of bromine as shown in Table 3 during the co‐pyrolytic treatment of TBBPA with twice the amount of Ca(OH)_2_ was discovered by Ali and coworkers^[201]^ through a holistic restriction of inorganic bromine both in the gaseous phase and pyrolytic oil. The captured inorganic bromine was completely fixed by the solid Ca(OH)_2_ in the form of CaBr_2_ and retained in the pyrolytic residue attributed to the solid‐liquid bromination process beyond the melting point of TBBPA in the low‐temperature ranges (200–300 °C).^[202]^ The thermo‐kinetic analysis coupled with the prediction of thermogravimetric data using ML approaches reinstate the findings.[^202^, ^203^] A debromination efficiency of 80.49 % was obtained during the co‐pyrolytic degradation of a mixture of novel BFRs [TBP, ATE and tetra‐bromo‐bisphenol A‐bis (2,3‐dibromo propyl ether)] with Ca(OH)_2_ between 25–500 °C.^[204]^ Interestingly, Gao and coworkers^[205]^ also found that Ca(OH)_2_ plays a vital role in capturing both organic and inorganic Br^−^, without affecting the product yield as the Ca and Br coordination could stretch and weaken the C−Br bond. Moreover, the CaO catalyst also outpaced the acidic catalysts by exhibiting an excellent removal of bromine with ∼100 % debromination efficiency during the catalytic pyrolytic effects comparison study of acidic and basic catalyst configurations over the arenes production and other volatiles from the co‐pyrolysis with flexible printed circuit boards waste.^[206]^ The HBr‐capturing capacity of Ca additives from brominated printed circuit boards in the recent studies[^207^, ^208^, ^209^] could be utilized in any thermal treatment of a brominated polymer, recommending the non‐release of the corrosive HBr gas into the atmosphere. Also, the recent BFR degradation studies tabulated in Table 3 endorse that any controlled thermal degradation technique using calcium additives could serve as a viable solution in mitigating the associated risk. As a futuristic perspective, it should include developing an advanced technique exploring a precise synergistic co‐pyrolysis, coupling bromine with non‐metals, and controlling the Br^−^ migration paths. These approaches should probably help in the mitigation of the toxic effects of BFRs during e‐waste recycling while potentially recovering valuable metals/non‐metals. These studies should also highlight the importance of developing an eco‐friendly, cost‐effective, and practical thermo‐catalytic technique for all BFRs degradation to address their environmental impact and potential health hazards.

## 3 Thermal Dehalogenation Approaches upon the Metal Surface

**Figure 9:**
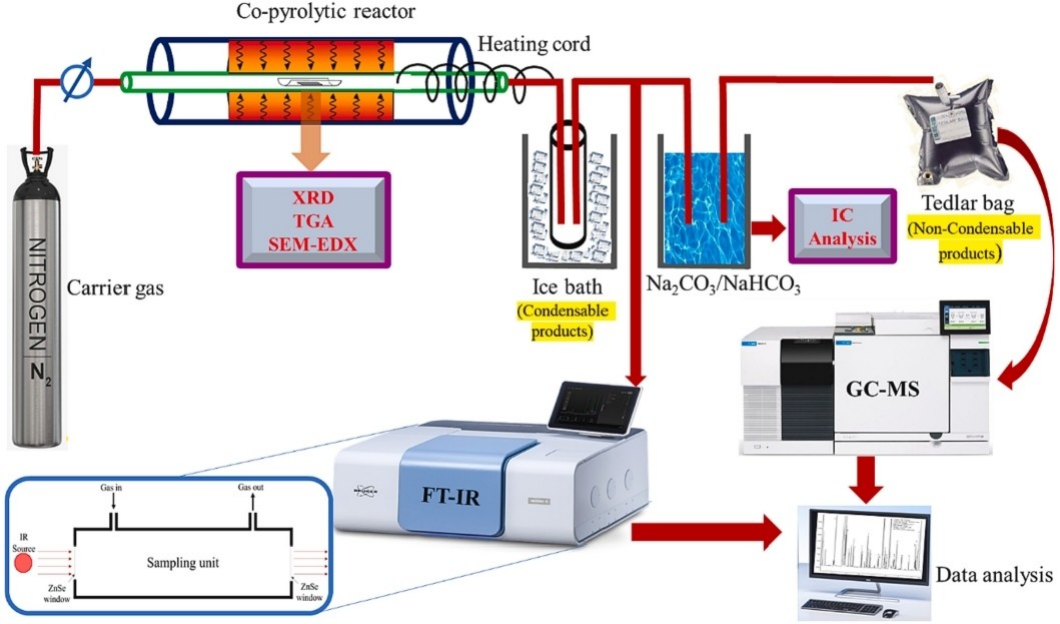
Experimental set‐up used for co‐pyrolytic studies. Reproduced with permission.^[201]^ Copyright © 2023, Elsevier Ltd.

The thermochemical conversion studies for the co‐pyrolysis of halogenated polymers with various additives (predominantly MOs) have been studied experimentally as shown in Figure 9. The co‐pyrolytic mixtures consisting of the target halogenated polymer and the selected additive are placed in the heating zone of the continuous flow pyrolytic reactor. The N_2_ carrier gas is flown at a constant flow rate of 80 ml/min. During the degradation, the carrier gas transports the gas emitted over the sample into multiple baths. The first is the ice trap where the condensable products are collected and the second is the sodium wash trap (Na_2_CO_3_/NaHCO_3_ solution) where the toxic acidic gases emitted from the halogenated polymer are captured. The gas emitted after the second bath is collected via a Tedlar bag and product analysis is administered using the gas chromatography‐mass spectrometer (GCMS). The condensable products in liquid form, which is also known as the pyrolytic oil, are analysed via GCMS. The hydrogen halides captured in the second bath are eventually taken for ion‐chromatography (IC) analysis to quantitatively analyze the dehalogenation efficiency. The gaseous products may also be subjected to infra‐red (IR) analysis to identify the molecular bonding involved. The pyro chars obtained are subjected to X‐ray diffraction (XRD) analysis, thermogravimetric analysis (TGA) and Scanning electron microscopy‐energy dispersive X‐ray (SEM‐EDX) analysis for characterizing the crystallinity, determining the elemental composition, quantifying the elements associated as well as mapping them out. The targeted temperature of analysis is done based on the TGA of the pure mixture wherein the degradation temperature window and degradation stages are defined. All the characterization studies involved are discussed in section 4.

### 3.1 De‐halogenation Mechanism based on DFT Calculations

Density functional theory (DFT) calculations offer an unmatched insight into chemical events that dictate the degradation of neat, halogenated compounds and their surface de‐halogenation pathways. In co‐pyrolysis of the halogenated molecules with the solid materials (mainly transition metal oxides), the latter act as halogen fixation agents, rather than as catalysts. A critical aspect in carrying out the DFT computations for a given system is to establish a benchmark of accuracy against relevant experimentally measured values comprising geometries, vibrational frequencies, thermo‐kinetic parameters, or band gaps.^[219]^ The underlying methodology and the computational framework are illustrated in several recent studies.[^220^, ^221^] With a pioneering very accurate theoretical framework and the emergence of powerful computational supercomputers, large molecular systems can now be investigated with astonishing accuracy. For gas phase reactions, energies can now be obtained with a sub kJ/mol accuracy. In surface‐assisted de‐halogenation reactions, the dehalogenation agent is typically represented as an extended surface or a cluster. The former offers a real representation of the material along one of the Miller indices while the latter provides an emphasis on the active sites that derive the de‐halogenation pathway. Insight acquired from DFT computations taps into four categories; adsorption energies, potential energy surfaces for the de‐halogenation process, transformation pathways of the metal oxides into oxyhalides and halides metallic surfaces, and micro‐kinetic surface models.^[33]^

**Figure 10:**
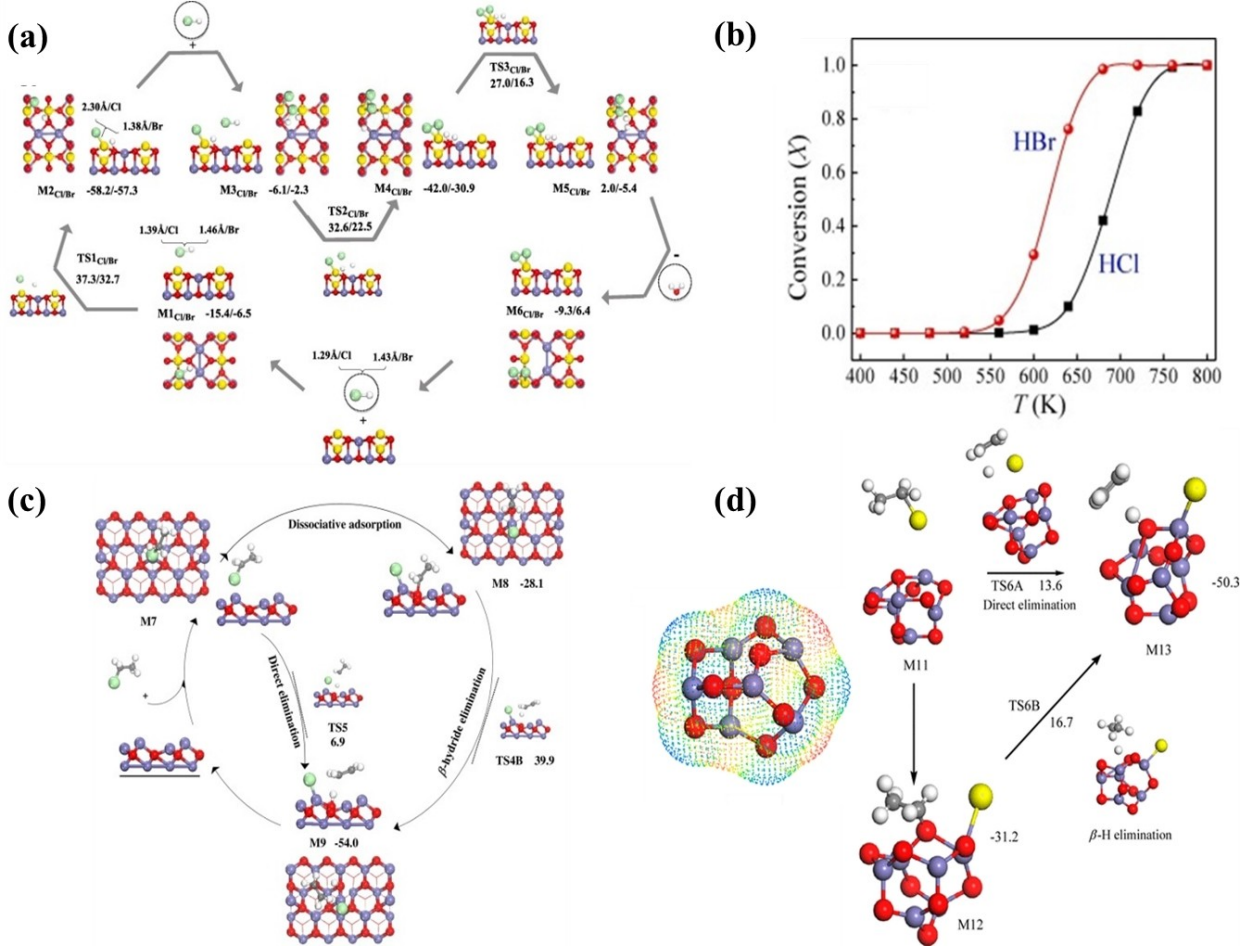
(a) Dissociative adsorption of HCl/HBr over franklinite; (b) computed conversions predicted by the constructed surface model; the dissociative dehalogenation mechanisms of (c) chloroethene over Fe_3_O_4_ (111) surface and (d) bromoethane over Fe_2_O_3_ cluster. All the values of energies are in kcal/mol. All the images are reproduced with permission.[^223^, ^225^, ^227^] Copyright © 2016, American Chemical Society. Copyright © 2019–2021, Elsevier Ltd.

As Table 4 shows, investigated halogenated compounds include hydrogen halides (HCl or HBr), and halogenated aliphatic and aromatic compounds while target additives mainly comprise oxides of copper, iron, lead, zinc and calcium. The latter represents the oxide phases in the electric arc furnace dust; a commonly utilized heterogenous waste in the co‐pyrolysis of halogenated waste materials.^[222]^ It shall be noted that DFT studies investigated the interaction of decomposition products from PVC and BFRs with transition metal oxides (mainly hydrogen halides and aliphatic C_1_‐C_3_ cuts), rather than with the halogenated polymer. We envisage that future studies will consider more representative large fragments of BFRs, PFASs, and PVC. Likewise, to the best of our knowledge, there are no DFT studies pertinent to the de‐fluorination capacity of experimentally tested materials such as transition metal oxides anchored on granulated activated carbon (GAC). As an illustrative example of de‐halogenation mechanisms derived by DFT computations, Figure 10(a) portrays the uptake mechanisms of HCl/HBr over franklinite, while Figure 10(b) depicts the corresponding surface‐assisted conversion of HBr/HCl.^[227]^ Likewise, Figure 10(c) and Figure 10(d) show dissociative dehalogenation mechanisms of chloroethene over the Fe_3_O_4_(111) surface^[225]^ and bromoethane over the Fe_2_O_3_ cluster^[223]^ respectively.

**Table 4:** A summary of selected DFT studies that investigated the de‐halogenation capacity of transition metal oxides.

Halogenated entity	Additives	Representation	Theoretical level	Remarks	Reference
HBr, aliphatic and aromatic brominated species	Fe_2_O_3_	Cluster	LDA‐PAW	PESs and kinetic parameters were computed.	^[223]^
HBr, HCl	ZnO	Surface	GGA‐PPE	A simplified kinetic model was provided for the uptake of hydrogen halides.	^[224]^
HCl, Chlorinated aliphatic	Fe_3_O_4_	Surface	GGA‐PW91	PESs and kinetic parameters were computed.	^[225]^
HCl, Chlorinated aliphatic	Fe_2_O_3_	Cluster	LDA‐PAW	Adsorption energies and reaction pathways were computed.	^[226]^
HBr/HCl, aliphatic and aromatic halogenated species	ZnFe_2_O_4_	Surface	GGA‐PW91	A micro‐surface kinetic model was developed.	^[227]^
HBr	Ca(OH)_2_	Cluster	LDA‐PAW	Transformation of Ca(OH)_2_ into CaBr_2_ is described.	^[201]^

During the thermal degradation of BFRs and PVC, most of their halogen content is transformed into hydrogen halides.^[33]^ As such, most relevant DFT studies have focused on the interaction of HCl and HBr with prominent transition metal oxides as a primary dehalogenation step. These hydrogen halides are emitted in the initial degradation steps via unimolecular pathways or through secondary abstraction reactions. As confirmed by XRD measurements, transition metal oxides are transferred into their corresponding halides upon their co‐pyrolysis with BFRs or PVC. As illustrated by DFT computations, hydrogen halides assume a chief role in this observed transformation. The governing mechanism entails three steps, subsequent uptake of HCl/HBr by the surface, intramolecular hydrogen transfer to form surface OH* and H_2_O* groups, and water desorption as shown in Figure 10(a). De‐halogenation of aliphatic cuts takes place via two competing mechanisms, surface‐assisted direct elimination of a hydrogen halide molecule or fission of the C‐halogen bond catalyzed by the surface followed by a *β*‐H as illustrated in Figure 10(a), (c) and (d). The metallic oxide bonds act as Lewis acid/base sites for the dissociative addition of hydrogen halides and aliphatic molecules.

### 3.2 De‐fluorination Mechanism

**Figure 11:**
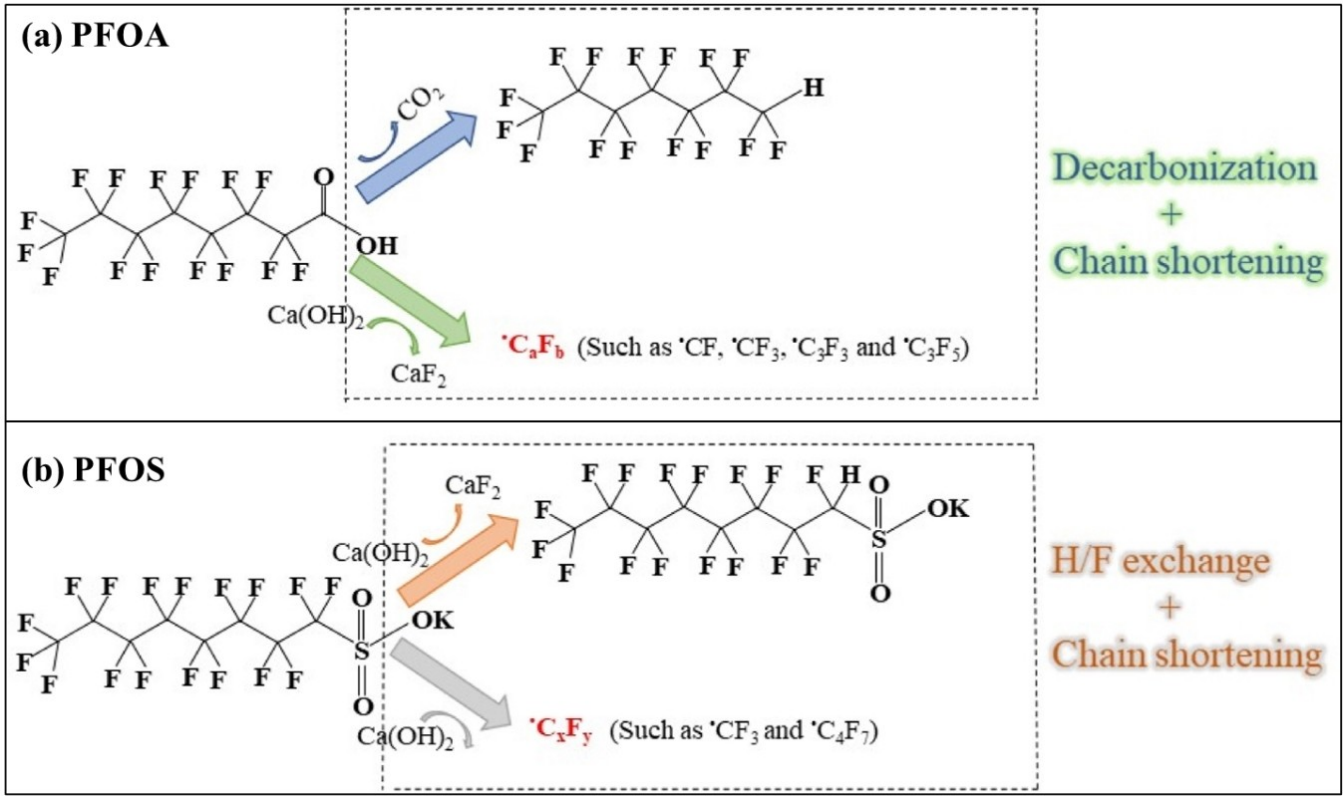
A defluorination mechanism was proposed using Ca(OH)_2_ for (a) PFOA and (b) PFOS. Reproduced with permission.^[73]^ Copyright *©* 2021, Elsevier Ltd.

A per‐fluorinated chain is principally an alkyl chain wherein all the H‐atoms are traded off with F‐atoms. The difference in the sulfonate head, carboxylate head and tail of the per‐fluorinated chain is attributed to their uniqueness from their hydrogenated counterparts. Though PFAS are predominantly found in water matrices, they could be present in soil too. Because of the intricacy of matrices and the abundance of intermediate and incomplete PFAS degradation derivatives, a comprehensive mix of targeted investigations is necessary to gain the most thorough PFAS degradation mechanism understanding. The thermal degradation mechanism studies of PFAS based on experiments suggested direct F‐elimination from PFCAs.^[228]^ Whereas various DFT studies suggested the 1° PFOA decomposition mechanism is C–COOH cleavage, while for GenX it is α‐elimination pathways.[^229^, ^230^] Another DFT study by Altarawneh and coworkers^[231]^ illustrated that the direct C−C β‐bond fission is preferred over the terminal C−C bond fission or F‐elimination thermodynamically due to the respective energies 162<232<245 kJ/mol resulting in the production of per‐fluoroalkenes. The initial homolytic cleavage of the parent PFAS molecule eventually leads to the perfluoroalkyl radicals formation, transforming into organo‐fluorine products of incomplete destruction (PIDs), including per‐fluoroalkenes via HF elimination.[^232^, ^233^] Metal oxides often exhibit both Lewis acidic and basic sites on their surfaces and hence are preferred for the catalytic de‐fluorination of PFAS due to their redox properties, thermal stability, reaction E_a_ reduction and thereby reducing energy consumption. The defluorination mechanism of PFAS during thermal treatment with Ca(OH)_2_ involves the core process of hydro‐defluorination, constituting the following steps: (i) lowering the decomposition temperature of PFAS from 425 °C to 350 °C, (ii) C−F bonds are converted to C−H bonds, and (iii) a step‐wise trimming of PFAS into linear short‐chain (C_4_‐C_7_) per‐fluoroalkyl carboxylic acids (PFCAs), elemental fluorine and gaseous by‐products such as hydrogen fluoride and cyclic per‐fluoroalkanes.[^73^, ^234^, ^235^] The activation of Ca(OH)_2_ catalytic surfaces is induced by thermal treatment providing even stronger reducing sites for PFASs; continued heating generates new surfaces for PFAS contact which aids in the PFASs molecules decomposition into shorter chain fluorinated radicals which is even more reactive than the parent PFASs molecules. Deng and coworkers^[73]^ proposed that PFOA would crumble as shown in Figure 11(a) into many ⋅C_n_F_m_ cuts such as ⋅CF, CF_3_, ⋅C_3_F_3_ and ⋅C_3_F_5_ together as a function of temperature. Due to the higher activity of the α‐location −CF_2_‐bonds for PFOS, Deng and coworkers^[73]^ proposed a substitution reaction therein as shown in Figure 11(b) adjacent to the sulfonic head. At 350 °C, Ca(OH)_2_ initiates the decomposition and hence the decomposed PFOS produces CaF_2_, due to the solid‐state interaction between PFOS and the former. The H_2_O released from Ca(OH)_2_ during CaO formation reacts with CaF_2_ generated to produce HF. Ca(OH)_2_ is also anticipated to trigger the PFOS decomposition in the generation of many ⋅C_n_F_m_ cuts including ⋅CF_2_, ⋅CF_3_ and ⋅C_4_F_7_ at a temperature lower than 425 °C (gasification temperature).^[236]^

### 3.3 De‐chlorination Mechanism

**Figure 12:**
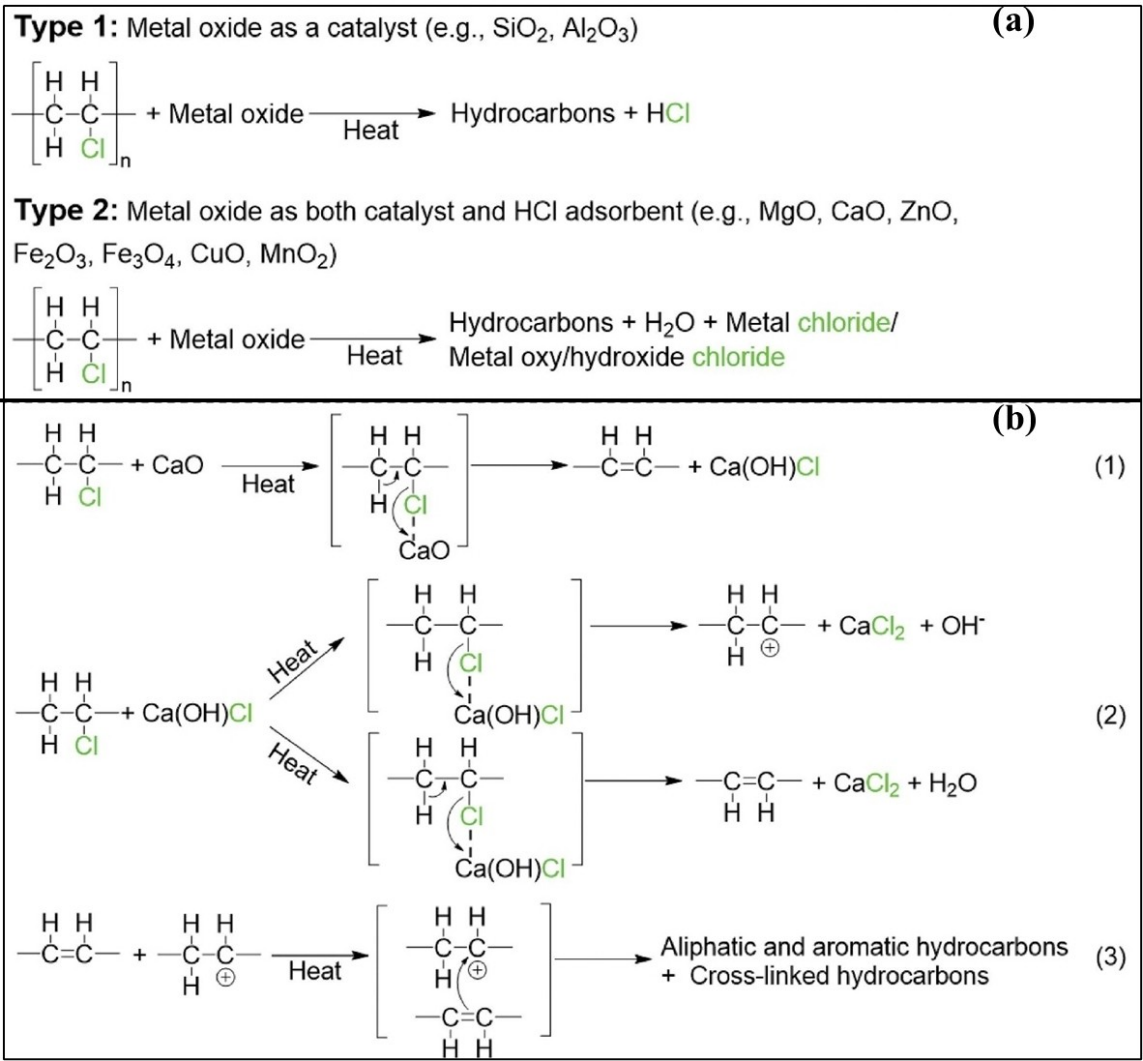
Proposed PVC thermal degradation pathways with (a) different metal oxides and specifically by (b) CaO. Reproduced with permission.^[100]^ Copyright *©* 2023, Elsevier Ltd.

Multiple processes are involved in the intricate process of thermally de‐chlorinating PVC. A non‐free‐radical pathway first forms conjugated polyene sequences, which subsequently react with HCl to generate radicals that extract H_2_ and make chloro‐allylic structures by autocatalysis, in which HCl is essential for accelerating the process; de‐hydrochlorination occurs in the first stage resulting in an approx. weight loss of 58 % at 250–350 °C.^[237]^ Thermo‐dehydrochlorination is mostly initiated at these allylic chlorine sites via various mechanisms such as molecular concerted reactions involving 1,2‐elimination, 1,4‐elimination, and 1,3‐rearrangement steps from allylic chlorine atoms catalyzed by HCl at low and moderate temperatures, are supported by molecular orbital simulations. Ionic and radical processes can also occur under specific conditions, with macro carbocations and macro radicals encouraging rapid degradation via different routes in the second stage that result in the creation of aromatic compounds and linear polyenes occurring at 350–525 °C. However, the radical reactions could probably predominate when HCl is present.[^238^, ^239^] However, with the energy barriers ranging from 167.4 to 243.3 kJ/mol, computational studies have shown that the allyl groups and the branched chains can lower the E_a_ for HCl elimination.^[239]^ Various additives, such as Hg, Ag and glass can affect the rate of autocatalytic de‐hydrochlorination. The thermal de‐hydrochlorination of PVC using metal oxides follows a two‐step mechanism. Initially, the chlorine free radicals form, followed by their reaction with the metal oxides to produce the respective metal chlorides thereby releasing oxygen‐free radicals. The effect of MOs on PVC degradation varies based on their acidity or basicity. The acidic oxides usually accelerate chlorine recombination with double bonds, while basic oxides inhibit this process. Whereas, Fe_2_O_3_ accelerates HCl elimination from PVC, with its effect depending on the concentration.^[240]^ The addition of ZnO to PVC alters the degradation kinetics by increasing one more decomposition stage instead of the three observed for pure PVC.^[128]^ The E_a_ for PVC de‐hydrochlorination was lowered by the presence of ZnO by ~10 kJ/mol. Chlorine abstraction on ZnO, the creation of zinc oxy/hydroxide chloride phases, and their subsequent breakdown are the mechanisms that eventually result in a notable decrease in the E_a_ for polyene thermal cracking.^[128]^ Certain metal oxides such as SiO_2_ and Al_2_O_3_ catalyze hydrochloride generation whereas others act as both catalysts and as HCl adsorbents producing the respective metal chlorides as proposed by Lu and coworkers^[100]^ as shown in Figure 12(a). During the thermal treatment (1) the calcium oxide interacts with the chlorine atom present in the PVC to yield calcium hydroxy chloride and polyene. The formed (2) calcium hydroxy chloride follows the interaction with chlorine in the thermal process to form calcium chloride, polyene, and an electrophilic complex eventually releasing water. The subsequent (3) thermal disintegrations and cyclization of polyene produce aliphatic hydrocarbons and arenes. Ensuing via electrophilic additions, the electrophilic complex orients towards the formation of cross‐linked hydrocarbons either by carbon‐chain polycondensation, by the cyclization via free radical's elimination, or by the intermolecular Diels‐Alder reactions as shown in Figure 12(b).[^100^, ^241^]

### 3.4 De‐bromination Mechanism

The thermal debromination mechanism of a selected legacy BFR compound such as tetrabromobisphenol A (TBBPA) might involve numerous pathways. However, the thermal treatment of brominated polymers launches a molecular Br‐enriched environment; at temperatures between 280–350 °C, the homolytic β‐scission of C_aromatic_<

**Figure 13:**
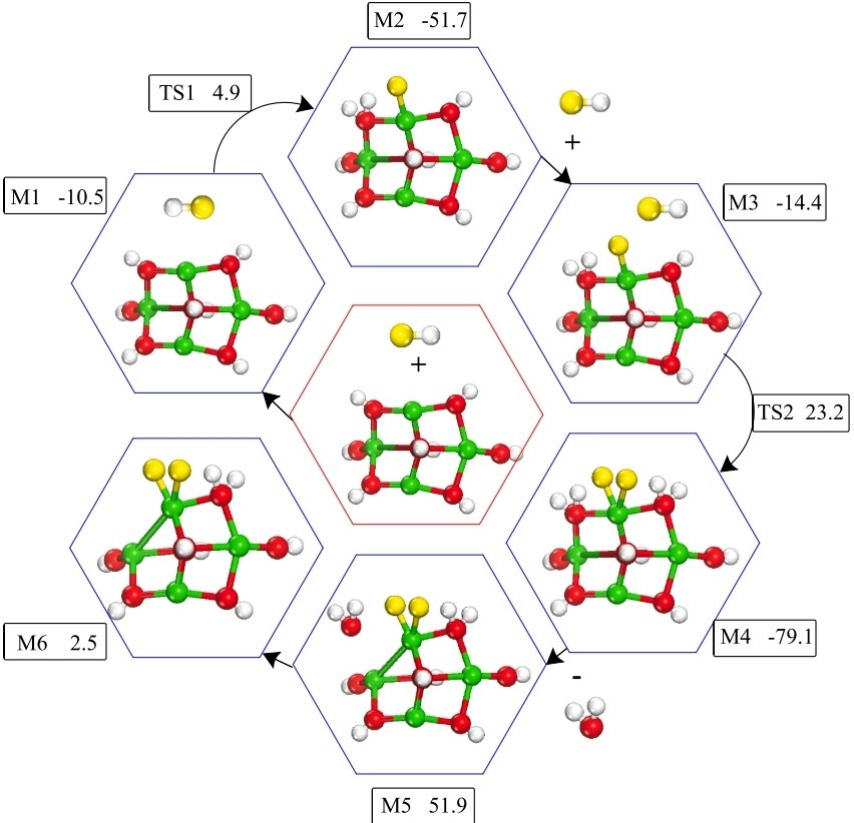
Pathways for thermo‐catalytic Ca(OH)_2_ transformation into CaBr_2_. Reproduced with permission.^[201]^ Copyright *©* 2023, Elsevier Ltd.

C‐>C bonds and Br_aromatic_, its condensation with the phenolic hydroxyl are preliminary processes which usually produce HBr, cut brominated phenols, and PBDD/Fs.^[201]^ The mechanisms could probably include direct pathways like debromination and the C−C bond cleavage and indirect pathways such as OH‐addition, Br‐substitution and H‐abstraction by singlet oxygen; producing OH‐adducts, Br‐substitution products, H‐abstraction products, and mostly brominated/de‐brominated products. In a gas‐phase decomposition, elimination of a methyl group is the dominant pathway in the unimolecular decomposition followed by the β C−H bond scission whereas in the bimolecular reactions with ⋅Br and ⋅CH_3_, abstraction of hydroxyl H atoms constitutes the most important pathway.[^242^, ^243^] By virtue of the metal oxides often exhibiting both Lewis acidic and basic sites on their surfaces, they can facilitate the surface adsorption and activation of BFR molecules, encouraging their subsequent debromination mechanism. The BFRs mechanism with metal oxides involves surface coordination between bromide and metal oxide surface, weakening the C−Br bond. The main pathways comprising the reaction between HBr and the metal oxide, direct elimination and dissociative adsorption coupled with β‐H elimination of organo‐bromines with metal oxides, and their deposition on the catalysts. The metallic oxide bonds act as Lewis acid/base sites for the dissociative addition of HBr molecules. The dissociative adsorption of HBr into adsorbed H and Br atoms was found to be catalyzed by the non‐polar ZnO(1010) surface.^[190]^ For the initial surface‐assisted uptake of the HBr, the mechanism involving water elimination through an intramolecular H transfer, resulting in the formation of metal bromides demands a trivial barrier of 4.9 kcal/mol (for Ca−O surface) as shown in Figure 13 and 8.2 kcal/mol (for Fe−O surface) only thereby initiating the bromination process coupled with the generation of –OH_2_ sites.[^201^, ^223^] An uncatalyzed bond fission demands a higher energy of 87.1 kcal/mol due to the relatively strong H−Br bond in HBr. The dehydrobromination of bromoethane occurs via a direct elimination one‐step mechanism over Fe_2_O_3_‐producing ethene, whereas acetylene formation involves a comparable reaction rate for dissociative decomposition and direct elimination pathways from vinyl bromide.^[223]^ The debromination of organics such as olefins and alkanes occurs through direct abstraction of Br by Fe surface sites, rather than through the dissociative adsorption leading to the H* and Br* surface sites generation.[^195^, ^244^] The direct surface‐mediated mechanism over C−Br bonds de‐brominates the bromoarenes and brominated aliphatics. Through DFT modelling, it was found that the Ca(OH)_2_ catalyst's surface‐assisted fission of aromatic C−Br bond in bromobenzene and the scission of the C−Br bond in the 2‐bromophenol incurred a trivial barrier of 5.3 kcal/mol and 4.7 kcal/mol respectively rendering swift surface‐assisted C−Br bond scission whereas the fission pertaining to the O−H bond was dubious due to its substantial energy barrier of 57.5 kcal/mol leading to the production of phenols.^[204]^ Similarly, the direct abstraction of bromine from an aromatic molecule took place via a trivial energy barrier of only 15 kJ/mol^[195]^ endorsing the bromine removal during the thermal treatment of BFRs.

## 4 Proposal of a Feasible Thermochemical Recycling Unit

**Figure 14:**
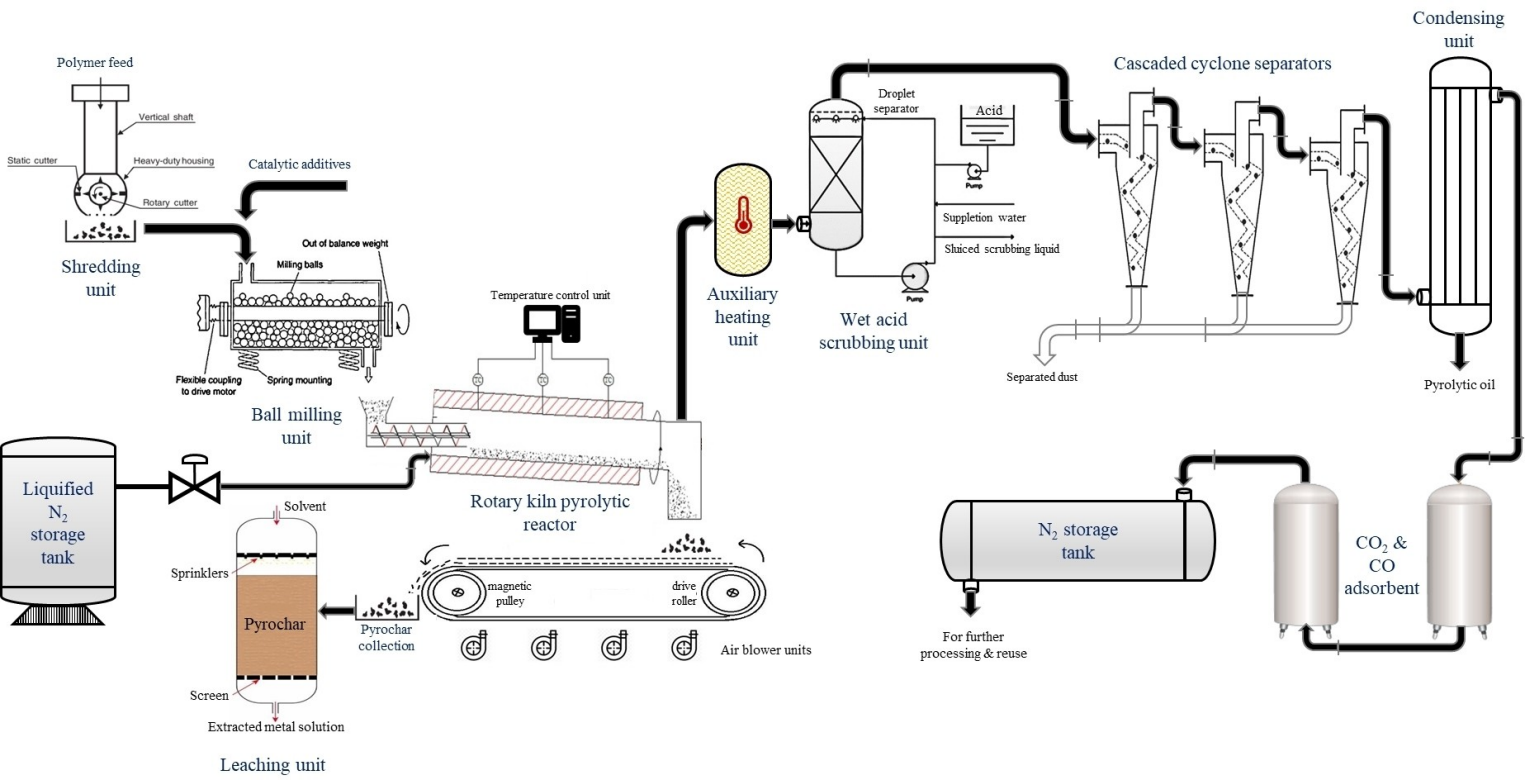
Proposed thermochemical recycling plant layout.

The thermochemical process such as co‐pyrolysis with metal oxides is a prospective opportunity for the recycling and valorization of halogenated polymeric wastes. Based on the recent TGA studies, the addition of metal oxides significantly reduces the E_a_ of the mixtures. ZnO appears to be an effective additive in PVC degradation,^[128]^ reducing the temperature and energy required for PVC decomposition but PbO and Fe_2_O_3_ weren't a good choice. MOs like ZnO, PbO, and Ca(OH)_2_ demonstrate varying levels of HBr capture efficiency coupled with E_a_ lowering during the co‐pyrolysis with BFRs.[^31^, ^190^, ^201^] The MOs such as CaO and Al_2_O_3_ have shown some potential for PFAS degradation, though these studies lacked thermo‐kinetic analysis.[^232^, ^245^] The GAC is believed to adsorb PFAS but eventually, it may lead to the formation of other volatile organic fluorine compounds. Table 5 provides some valuable insights into the potential of various MO studies as additives for the thermal degradation of various halogenated polymers by the recent TGA analyses. The lack of crucial thermo‐kinetic parameters hinders the in‐depth understanding of the degradation mechanisms. Future research focusing on the detailed thermo‐kinetic study accompanied by the comprehensive characterization of the degradation products is crucial for deriving an optimal mixing ratio, which could probably lead to the design of an effective and sustainable waste management solution. Based on the comparison study from Table 6, each method has trade‐offs between various factors such as efficiency, environmental impact, cost and applicability. The biological, subcritical, photocatalytic, and electrochemical processes are more eco‐friendly, but all of them are slow or require high initial investments and specialized equipment. However, the chemical and thermal processes have an upper hand over them due to their efficiency and scalability, but generate undesirable by‐products. Though thermo‐catalytic processes are energy‐intensive, the high efficiency, eco‐friendliness and versatility in addition to valuable by‐product generation give it an upper edge over the other treatment processes. The reductive reactions in the dehalogenation process are also endorsed by the literature review (Figure 3) directing toward a catalytic approach. Figure 14 shows a tentative plant layout for processing the halogenated polymeric wastes exploiting suitable catalytic additives such as transition metal oxides. After passing the waste polymers through the shredding unit and combining them with the catalytic additives, a ball milling unit ensures the proper mixing of the additives to exploit the synergy between them. The milled entities are fed via a hopper and a screw feeder into a rotary kiln pyrolytic reactor wherein the pyrolytic gases are channeled through heated pipelines into a condensing unit. The halogenated acids are scrubbed using an alkaline medium such as sodium hydrogen carbonate/sodium carbonate solution to make sure the pyrolytic oil is free from any toxic halogenated acids. The non‐condensable gases are further transferred into CO_2_ and CO adsorbent units containing amine‐functionalized solid material adsorbents or amine‐decorated metal‐organic framework (MOF) material so that the carrier N_2_ gas can be utilized for further processing or reuse.[^246^, ^247^] The pyrochar collected from the conveyor belts beneath the tail end of the rotary kiln could be used for metal recovery and extraction via further leaching. The leaching process of the pyrochar could be conducted using water as a leaching agent, maintaining a pH of 5–6, wherein the metal halides dissolve effortlessly within an hour.^[142]^ The pH of the leaching agent plays an important role^[139]^ in selectively separating/ recovering different metals when EAFD or a mixture of MOs are used as catalysts.

**Table 5:** A summary of the recent TGA studies (within 3 years) of the halogenated polymers mixed with different entities.

Mixing entity	Mixing ratio (Entity : Polymer)	Carrier gas	Degradation stages	Major findings	References
Per‐ and poly‐fluoroalkyl substances (PFAS)					
Pd/Al_2_O_3_	2 : 1	N_2_	2	PFOA mineralization was effective, thermo‐kinetic analysis wasn't done.	^[232]^
GAC	Unspecified	N_2_	2	GAC's contribution in the HFPO–TA degradation and defluorination after being adsorbed, no thermo‐kinetics study.	^[248]^
CaO	4 : 1	Dry air	3	PFAS‐laden resins held the PFAS compounds well above their boiling points, with no thermo‐kinetic studies done.	^[245]^
GAC	400 mg (GAC): 50 mL (PFOA solution)	N_2_	3	PFOA decomposition yielded >45 volatile organic fluorine products due to adsorption, no thermo‐kinetic studies.	^[249]^
Pt/Al_2_O_3_	2 : 1	N_2_	3	Less efficacy in PFOA mineralization, thermo‐kinetic study missing.	^[232]^
Polyvinyl chloride (PVC)					
ZnO	0.65 : 1	N_2_	4	HCl captured was ∼46.3 % at 50 K/min heating rate, decreasing the T_onset_ by 58 °C, E_a_ reduced by ~10 kJ/mol.	^[128]^
EAFD	1 : 1, 1 : 2 and 1 : 3	N_2_	2	Chlorine fixation capability of EAFD was 48.4 % for long cylindrical powders, HCl capture dropped from 78.9 % to 48.2 % and 34.5 % respectively for 1 : 1, 1 : 2 and 1 : 3 ratios.	^[250]^
PbO	1.79 : 1	N_2_	3	E_a_ increased by 25 kJ/mol, and the onset of PVC de‐HCl increased by 20 °C, portraying an inhibition effect.	^[251]^
PbO	0.56 : 1	N_2_	3	Acceptable HCl capture efficiency (45.57 %), thermo‐kinetic analytics not done.	^[31]^
EAFD	1 : 1, 1 : 2 and 1 : 3	O_2_	3	Prevented Fe chlorination while ZnCl_2_ and PbCl_2_ were formed, suppressed the iron extraction by oxidizing Fe_3_O_4_ into stable Fe_2_O_3_.	^[250]^
Fe_2_O_3_	0.427 : 1	N_2_	6	E_a_ increased by ~77 kJ/mol, an inhibition effect.	^[252]^
CaO	1 : 1	N_2_	2	Total chlorine in the pyrolytic oil was 62 ppm and 6.3 ppm of organic chlorine.	^[253]^
Fe_3_O_4_	0.46 : 1	N_2_	5	E_a_ increased by ~55 kJ/mol due to the inhibition effect.	^[252]^
Brominated fire retardants (BFRs)					
ZnO	1:0.345	N_2_	2	Tetrabromobisphenol A 2,3‐dibromopropyl ether co‐pyrolysis generated 249.75 mg/g HBr, thermo‐kinetic analytics not done.	^[190]^
Ca(OH)_2_	2 : 1	N_2_	3	The activation energies for the mixture with TBBA were reduced by ~50 kJ/mol, ▵G values were positive.	^[202]^
Fe_2_O_3_	1 : 1	O_2_	2	Lowered E_a_ for tribromophenol by ~20 kJ/mol, ‐▵S, +▵G.	^[196]^
ZnFe_2_O_4_	1:0.258	N_2_	4	Tetrabromobisphenol A diallyl ether co‐pyrolysis generated 145.86 mg/g HBr, thermo‐kinetic analytics not done.	^[190]^
PbO	0.714 : 1	N_2_	3	Tetrabromobisphenol A diallyl ether co‐pyrolysis showed better HBr capture efficiency (80.04 %), thermo‐kinetic analytics not done.	^[31]^
Ca(OH)_2_	2 : 1	O_2_	3	E_a_ for the mixture with TBBA were reduced by ~74 kJ/mol, +▵G.	^[202]^
ZnFe_2_O_4_	1:0.34	N_2_	4	Tetrabromobisphenol A 2,3‐dibromopropyl ether co‐pyrolysis generated 59.5 % lesser HBr, thermo‐kinetic analytics not done.	^[190]^
Fe_2_O_3_	1 : 1	N_2_	2	Lowered E_a_ for tribromophenol by ~7 kJ/mol, ‐▵S, +▵G.	^[196]^
Al_2_O_3_	2 : 1, 1 : 1 and 1 : 2	N_2_	2	E_a_ for the bulk non‐metallic fractions was higher around 9.41–94.41 kJ/mol, with high thermodynamic stability.	^[194]^
ZnO	1:0.261	N_2_	3	Tetrabromobisphenol A diallyl ether co‐pyrolysis exhibited lower HBr emission (<8 %), thermo‐kinetic analytics not done.	^[190]^

**Table 6:** Comprehensive comparison table portraying the merits and demerits of various dehalogenation processes.

Dehalogenation process	Merits	Demerits	Comparison
Chemical process	Better dehalogenation rate and large‐scale application efficiency.	Harsh reaction conditions requirement and toxic residues.	Often less selective, less environment friendly and harsh conditions in comparison to other processes.
Biological process	Environmentally benign and sustainable, mild operating conditions.	Extremely slower process, suitable for certain compounds only.	Though environment friendly, comparatively slower degradation rate and limited applicability.
Thermal process	Very intuitive and wide applicability of compounds with acceptable efficiency, no special chemicals required.	Heavy energy consumption and toxic by‐product formation (oxidative treatment exempted).	Comparatively energy‐intensive and more complex by‐product generation.
Subcritical methods	Environment‐friendly solvents, selective cum enhanced reactions, improved mass transfer and highly efficient.	High initial investment, operational complexity and risk of handling high‐pressure system.	Comparatively more control over reaction conditions and associated risk of implementation.
Fenton's process	Strong oxidizing capability, lower cost, versatility, simple execution.	Acidic pH required, iron sludge by‐product, catalyst deactivation, limited application.	Less‐effective for highly recalcitrant compounds and comparatively very low pH requirement.
Electrochemical process	Highly selective, precisely controlled, minimal secondary products, ambient temperatures and pressures operation.	Specialized equipment required, costly initial set‐up, expensive catalysts.	Though eco‐friendly, comparatively complex set‐up and higher electrical energy consumption.
Photocatalytic process	Utilizes renewable energy, mild reaction conditions, high selectivity for certain halogens, highly eco‐friendly.	UV light availability, energy‐intensive, very expensive photo‐catalysts, slow reaction.	Though eco‐friendly and utilizes solar energy, comparatively slower reaction rate and specialized equipment are required.
Thermo‐catalytic process	Better efficiency, versatility, energy recovery potential, non‐specialized catalyst.	Significant energy consumption, catalyst pre‐treatment and catalyst deactivation.	Though energy‐intensive, comparatively faster reaction rate, wide applicability, pyro oil production with fewer by‐products, multiple industry waste utilization.

## 5 Characterization Techniques

**Figure 15:**
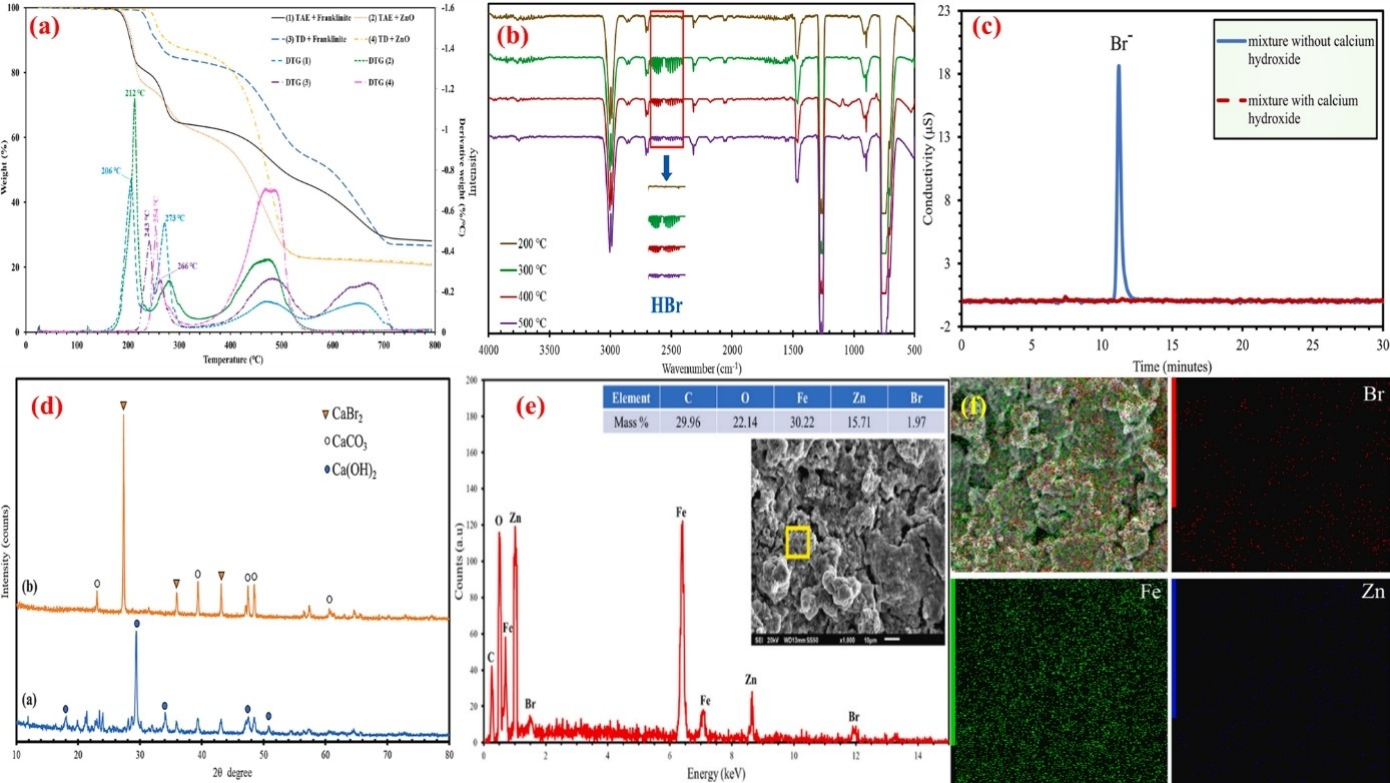
Various characterization techniques adopted in debromination studies; (a) TGA, (b) FTIR, (c) IC, (d) XRD, (e) SEM and (f) EDX analyses. All the images are reproduced with permission.[^190^, ^201^, ^204^] Copyright *©* 2023, Springer Nature. Copyright *©* 2023–2024, Elsevier Ltd.

Characterization techniques for any halogenated polymeric study can be initiated with TGA as it is an invaluable tool to study thermal stability, decomposition kinetics, compositional analysis, moisture and volatiles content, polymeric matrix filler content, oxidative stability and degradation behaviour of the polymers. The TGA provides valuable insights (Figure 15 (a)) into the thermal behaviour of a halogenated polymer, enabling researchers to make informed decisions about their degradation temperature profile and selection of the required temperature for the degradation study. A GCMS identifies qualitatively or quantitatively the degradation products from the halogenated polymers. According to the studies conducted by Ali and coworkers,^[201]^ the GCMS analysis at different temperature ranges for the target mixture revealed the absence of brominated compounds in the degraded products during the co‐pyrolytic treatment with Ca(OH)_2_, transforming them into *n*‐alkanes interestingly. We could achieve a better understanding of the chemical events taking place during the degradation, which could also help in the mechanistic investigations if we could examine the types and quantities of the degradation products in detail.^[204]^ Another method we could utilize for studying the breakdown of halogenated polymers would be the Fourier Transform Infrared Spectroscopy (FTIR) analysis, from the vibrational frequencies of the chemical bonds within the polymer. It is claimed that quantification of the degradation products can also be done from FTIR by measuring the intensity of their absorption bands such as C=O groups, OH groups, C−O−C antisymmetric vibration, =C−H aromatic bending, C−X bonds (where X=F, Cl, Br) stretching vibrations due to their strong dipole moment. Also, the hydroperoxides (OOH) which are often associated with the polymer oxidation. The stretching vibrational peaks of the H−Cl bond and H−Br bond (as shown in Figure 15 (b)) are found at the wavelengths of 3100–2650 cm^−1^ and 2800–2300 cm^−1^ respectively^[31]^ whereas the primary absorption band for H−F bond typically falls within the range of 4000–3900 cm^−1^ and mostly in the near‐infrared (Near‐IR) region based on the simulations by Liu and coworkers.^[254]^ The doublet peaks series associated with hydrogen halides such as HF, HCl and HBr alternatively cause the transmittance to rise and fall to the baseline. This is a diatomic molecule's characteristic rovibrational transitions within the ground and the first excited vibrational state of the molecule validated upon the National Institute of Standards and Technology (NIST) library basis. The solid FTIR analysis of the pyrochar could be utilized to compare the functional groups retained even after the thermal treatment of the halogenated polymers. The IC is an analytical technique that could be used to analyze and quantify the ionic species of the halogens released during the thermal treatment of the polymer when dissolved in a suitable solvent such as sodium wash^[190]^ to capture the HX gases (where X=F, Cl, Br). The retention time for each ionic species is unique, thereby enabling the researcher to quantify the target ions F^−^, Cl^−^ and Br^−^ as shown in Figure 15(c). The pyrochar can be subjected to XRD; a valuable technique for studying the structural changes that occur in halogenated polymers during co‐pyrolysis. It can provide perceptions into the crystallinity, crystallite size, and crystal structure of the new products formed during the mineralization of the halogens, which are retained in the pyrochar. The formation of CaBr_2_ minerals in the pyrochar during the co‐pyrolysis of TBBPA with Ca(OH)_2_ was validated by Ali and coworkers^[201]^ using the XRD technique as shown in Figure 15(d). Scanning Electron Microscopy (SEM) as a surface characterization tool could be used for the visualization of the surface morphology and other microstructural changes that occur during degradation. From the size, shape, and distribution of these features, we can quantify the extent of degradation and track its progression over time. Usually, SEM analysis is coupled with Energy‐Dispersive X‐ray Spectroscopy (EDX) as researchers can determine the elemental composition of the polymer surface and identify the presence of foreign elements (if any) and validate the degradation products as shown in Figure 15(e) and Figure 15(f). The energy of the characteristic X‐rays emitted by the elements present in the sample, such as C, O, F, Cl and Br will be directly related to the atomic number of the respective element with Kα and Lα values, which allows for the precise elemental identification.[^31^, ^204^] Similarly, certain SEM is usually coupled with Electron Backscatter Diffraction (EBSD) helping us in the analysis of the crystallographic orientation of the halogenated polymeric chains, which can provide a better understanding of the effect of degradation on the polymer‘s crystalline structure. X‐ray photoelectron spectroscopy (XPS) is another surface characterization tool that could be utilized for understanding the chemical changes that occurred in the pyrochar during the halogenated polymer degradation providing detailed information about the elemental composition and their chemical states as well^[255]^ differentiating between carbon atoms in various bonding environments (C−C, C−H, C=O, C−O−C, etc…) and a deep profiling of the degraded entities, allowing scholars to gain insights into the mechanisms and kinetics of degradation.

## 6 Conclusion

Halogenated polymers are ubiquitous and have become an integral part of everyday life. The waste associated with these polymers is a menace to waste handlers due to its persistent nature as it has serious environmental trade‐offs. While traditional treatment methods are ineffective, AOPs, sonochemical degradation, thermal treatment, and emerging biological treatments offer promising solutions, though each treatment comes with its own set of advantages and challenges. Though AOPs are promising technologies and are effective in breaking down a wide range of PFAS compounds, the high energy demand and operational costs along with the toxic by‐product formation could be challenging. All the chemical treatment techniques of chlorinated polymers end up in the production of corrosive HCl gas and no technique arrests these free chlorine radicals for restricting their atmospheric release. The recent photochemical degradation techniques show potential for BFR removal from brominated polymers, but further research is needed to address the formation of degradation products and optimize the treatment conditions for large‐scale implementation. Albeit biological treatments offer a greener approach for these halogenated wastes, the time consumed for the process is several weeks/ months and requires incubation at specific conditions in specific growth mediums. Hybrid methods, which combine multiple techniques, could enhance the overall efficiency of degradation, offering a promising approach to achieving a comprehensive degradation of the halogenated wastes. Metal oxides often exhibit both Lewis acidic and basic sites on their surfaces and hence are preferred for the catalytic de‐fluorination of PFAS due to their redox properties, thermal stability, E_a_ reduction and thereby reducing energy consumption; hence preferred for the co‐pyrolytic catalytic dehalogenation. While co‐pyrolysis with metal oxides currently offers a preferable solution for halogenated polymeric waste, its environmental trailmark remains a concern due to the associated products generated. Dehalogenation coupled with halogen mineralization provides an amicable solution to the concern. The co‐pyrolysis with metal oxides containing calcium additives currently epitomizes the most candid solution for halogenated polymeric waste due to: (i) the hydrofluoric acid neutralization propensity with milk of lime exhibiting higher mineralization efficiency with CaO and Ca(OH)_2_ promoting defluorination at temperatures <400 °C, (ii) the limiting reaction of the calcium hydroxy chloride formation tendency during the PVC thermal treatment with CaO, and (iii) solid‐liquid bromination process at low‐temperature ranges (200–300 °C) in capturing HBr while using Ca(OH)_2_ for combating BFRs. To move towards a sustainable dehalogenation approach, future research should focus on engineering genetically modified enzymes efficient enough to increase microbial degradation techniques, in addition to optimizing chemical processes to achieve valuable yields, capturing toxic emissions and reducing energy consumption. Continued research and innovation are essential to develop a safer, more efficient, cost‐effective and scalable method combining multiple degradation techniques utilizing nanotechnology, which could be pivotal in achieving a sustainable approach for a circular economy.

## Biographical Information

*Mohamed Shafi Kuttiyathil having a rich industrial exposure after graduating with B.Tech in Chemical Engineering degree from Calicut University (India), attained his Master's degree in 2020 from UAE University. Currently he is pursuing his PhD in Chemical Engineering at UAE University. He probed into the catalytic pyrolysis for e‐waste recycling focussing on novel brominated fire retardants. He expanded his research into dehalogenation studies concentrating on chlorinated polymers and further into the defluorination prospects. He is also associated in thermo‐catalytic biomass valorization projects*.

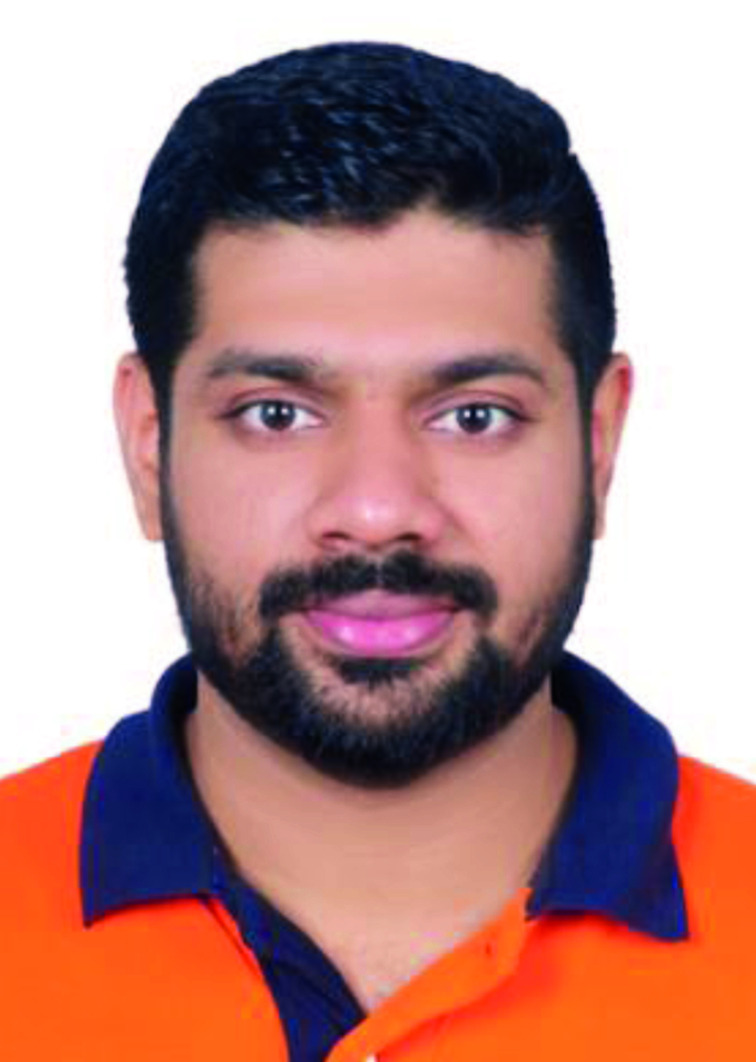

## Biographical Information

*Dr. Labeeb Ali holds a Ph.D. in Chemical Engineering from United Arab Emirates University (UAEU), UAE. He obtained Master of Science in Chemical Engineering from UAEU. He is currently a Postdoctoral fellow at UAEU. With over several research and review articles published in high‐impact journals, he has made extensive contributions to the field of chemical engineering. His active research areas include thermochemical conversions (combustion, pyrolysis and gasification), biomass valorization, halogenated pollutants, waste management, heterogeneous catalysis, energy conversion, and environmental pollutants as well as he drives in developing novel materials for energy and environmental related fields targeting the circularity and sustainability*.

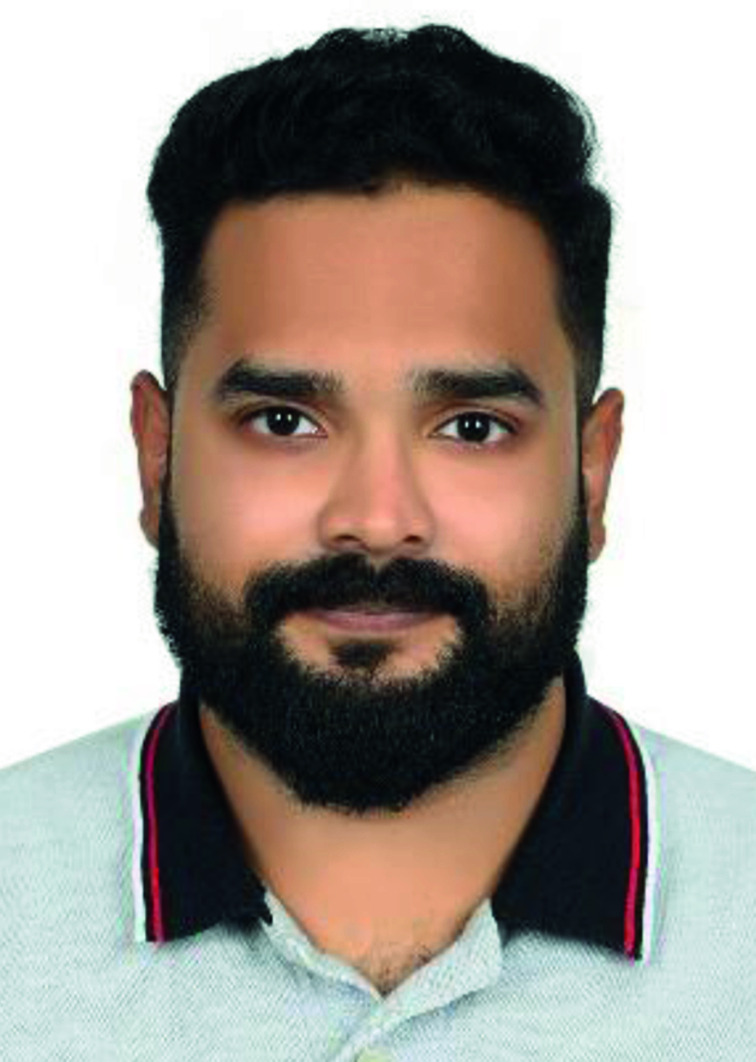

## Biographical Information

*Prof. Mohammednoor Altarawneh received his Ph.D. in computational physical chemistry from the University of Newcastle, Australia, in 2008. He is currently a professor of chemical engineering at the United Arab Emirates University. His research focuses on combustion kinetics, heterogeneous catalysis, and formation of halogenated pollutants*.